# Supplementary material for: Sulfur isotopes as a proxy for human diet and mobility from the preclassic through colonial periods in the Eastern Maya lowlands
Source: PLoS One. 2021 Aug 12;16(8):e0254992. doi: 10.1371/journal.pone.0254992 (PMC8360522; doi:10.1371/journal.pone.0254992)
Supplement: S1 Text — (DOCX) [file pone.0254992.s003.docx]

**S1 Text: Temporal assignments for directly dated burials**

Temporal assignment for burials in this study was determined based on contextual information recovered during excavation. Additionally, a total of 37 individuals have associated AMS ^14^C dates, which were analyzed using different aliquots of the same sample used for carbon, nitrogen, and sulfur described below. All dates were calibrated in OxCal v 4.4 (Bronk Ramey 2009) using the IntCal20 Northern Hemisphere atmospheric curve (Reimer et al. 2020). They are reported as 2σ calibrated ranges in cal year CE. Given the proximity of eastern Lowlands sites to different rivers and streams, and the likelihood of some amount of riverine food in the ancient Maya diet, an unquantified freshwater reservoir effect (Rf) may impact some or all the directly dated skeletons (see Hoggarth et al. 2014).

**Table S1.1:** Calibrated AMS ^14^C dates reported by Hoggarth et al. n.d. for samples analyzed in this study.

| **Sample ID #** | **Provenience** | **Lab ID** | **2**σ **range**  **(cal CE)** | **Citation** |
| --- | --- | --- | --- | --- |
| BKP09 | M-209, Burial 2 | UCIAMS-155972 | 415-540 | Hoggarth et al. n.d. |
| BKP11 | M-209, Burial 4 | UCIAMS-155973 | 420-540 | Hoggarth et al. n.d. |
| BKP15 | M-96, Burial 4 | PSU-1061 | 590-655 | Hoggarth et al. n.d. |
| BKP16 | M-99a, Burial 99N-1 | UCIAMS-132231 | 10-130 | Hoggarth et al. 2014:Table 1 |
| BKP29 | Bedran Group, Str. 2, Burial 2 | UCIAMS-155974 | 675-775 | Hoggarth et al. n.d. |
| BKP35 | M-102, Burial 1 | UCIAMS-155975 | 680-750 | Hoggarth et al. n.d. |
| BKP41 | North Caracol Farm M-12, Burial 1 | UCIAMS-172438 | 1230-1285 | Hoggarth et al. n.d. |
| BKP47 | Plaza B, Burial 4-1 | UCIAMS-172439 | 685-880 | Hoggarth et al. 2021 |
| BKP48A | Plaza B, Burial 4-2 | PSU-1698 | 705-955 | Hoggarth et al. 2021 |
| BKP52 | Grp B, Plaza B, Burial 3 | PSU-1838 | 705-880 | Hoggarth et al. 2021 |
| BC01 | Ledge 11, Surface collection north | PSU-1524 | 705-885 | Hoggarth et al. n.d. |
| CHP02 | Plaza B, EU-13 Burial 1 | UCIAMS-164842 | 435-585 | Ebert et al. 2019:Table 2 |
| CHP15 | Str. B4 (1 sub) | UCIAMS-151860 | 660-775 | Ebert et al. 2019:Table 2 |
| CHP16 | Str. B4, Lvl. 5, Burial 1 | UCIAMS-164844 | 655-775 | Ebert et al. 2019:Table 2 |
| CHP19 | Str. C2, Burial 1 | UCIAMS-166048 | 770-895 | Ebert et al. 2019:Table 2 |
| CHP21 | Plaza B Op. 4a Burial 1 | UCIAMS-166049 | 435-575 | Ebert et al. 2019:Table 2 |
| CHP24 | Plaza G, Unit 51, Level 2 | UCIAMS-166050 | 1660-1950 | Ebert et al. 2019:Table 2 |
| TLK08 | Tolok Group, Str. 14/15, Burial 8 | UCIAMS-164851 | 125-230 | Ebert et al. 2019:Table 2 |
| TLK10 | Tolok Group, Str. 14/15, Burial 10 | UCIAMS-151861 | 20-205 | Ebert et al. 2019:Table 2 |
| ZBN01 | Zubin Group, Str. A1, Burial A1-B/1 | UCIAMS-151862 | 595-660 | Ebert et al. 2019:Table 2 |
| ZBN13 | Zubin Group, Str. C9-6th, Burial C9-B/1 | UCIAMS-166059 | 670-820 | Ebert et al. 2019:Table 2 |
| ZTZ01 | Zotz Group, Str. B2, Burial 2-B/1 | UCIAMS-164852 | 700-880 | Ebert et al. 2019:Table 2 |
| ZTZ08 | Zotz Group, Plaza Unit 7 Burial 4 | UCIAMS-166056 | 425-540 | Ebert et al. 2019:Table 2 |
| ZTZ11 | Zotz Group, Str. B2, Burial 2-B/4 | UCIAMS-166057 | 430-545 | Ebert et al. 2019:Table 2 |
| ZTZ12 | Zotz Group, Str. B2, Burial 2-B/5 | UCIAMS-166058 | 240-345 | Ebert et al. 2019:Table 2 |
| ZTZ3b | Zotz Group, Str. B2, Burial 2-B/3 Indiv 2 | UCIAMS-166055 | 655-775 | Ebert et al. 2019:Table 2 |
| LWD08 | Courtyard 1, Burial 1 | PSU-1696 | 770-995 | Hoggarth et al. n.d. |
| LWD09 | Rockshelter 1 Burial 1 | PSU-1697 | 1300-1405 | Hoggarth et al. n.d. |
| PH02 | PKH-Burial 2A-1 | PSU-1288 | 650-775 | Hoggarth et al. n.d. |
| PH03 | PKH-Burial 2A-2 | PSU-1289 | 640-760 | Hoggarth et al. n.d. |
| PH04 | PKH-Burial 2A-3 | PSU-1290 | 640-775 | Hoggarth et al. n.d. |
| PH06 | PKH-Burial 4A-1 | PSU-1292 | 655-775 | Hoggarth et al. n.d. |
| PH10 | PKH-Burial 4A-3 Ind.G. | PSU-1294 | 665-775 | Hoggarth et al. n.d. |
| PH12 | SU61 Midden Cluster 6 | PSU-1347 | 665-825 | Hoggarth et al. n.d. |
| XUN05 | Str. A9 Burial 2 (Tomb) | PSU-1695 | 670-775 | Awe et al. 2019 |

**References**

Awe JJ, Helmke C, Slocum D, Tilden D. Let’s Talk of Graves, Eccentrics, and Epitaphs: The Socio-Political Implications of Recent Discoveries on Structure A9 At Xunantunich, Belize. Res. Rep. Belizean Archaeol. 2019; 16: 57-74.

Bronk Ramsey C. Bayesian Analysis of Radiocarbon Dates. Radiocarbon 2009; 51(1):337-360. doi: 10.1017/S0033822200033865.

Ebert CE, Hoggarth JA, Awe JA, Culleton BJ, Kennett DJ. The Role of Diet in Resilience and Vulnerability to Climate Change among Early Agricultural Communities in the Maya Lowlands. Curr. Anthropol. 2019; 60(4): 589-601. doi: 10.1086/704530.

Hoggarth JA, Culleton BJ, Awe JJ, Helmke C, Lonaker S, Davis JB, Kennett DJ. Building high-precision AMS ^14^C Bayesian models for the formation of peri-abandonment deposits at Baking Pot, Belize. Radiocarbon 2021. doi: 10.1017/RDC.2021.30.

Hoggarth JA, Culleton BJ, Awe JJ, Kennett DJ. Questioning Postclassic Continuity at Baking Pot, Belize Using Direct AMC ^14^C Dating of Human Burials. Radiocarbon 2014; 56(3): 1057-1075. doi: 10.2458/56.18100.

Reimer P, Austin W, Bard E, Bayliss A, Blackwell P, Bronk Ramsey C, et al. The Intcal 20 Northern Hemisphere Radiocarbon Age Calibration Curve (0–55 CAL kBP). Radiocarbon 2020; 1-33. doi: 10.1017/RDC.2020.41.
