# Supplementary material for: Sulfur isotopes as a proxy for human diet and mobility from the preclassic through colonial periods in the Eastern Maya lowlands
Source: PLoS One. 2021 Aug 12;16(8):e0254992. doi: 10.1371/journal.pone.0254992 (PMC8360522; doi:10.1371/journal.pone.0254992)
Supplement: S2 Text — (DOCX) [file pone.0254992.s004.docx]

**S2 Text: Stable Isotope Sample Preparation and Measurement Methods**

**Collagen Extraction**

For all samples, cortical bone was preferentially sampled when available to maximize collagen yield. Approximately 1000 mg of dry bone from each sample were cleaned of adhering sediment with an X-acto® blade. Bone collagen was extracted and purified using the modified Longin (1971) method with ultrafiltration (Brown et al. 1988). Samples were demineralized for 24–48 hours in 0.5 N HCl at 5 °C, followed by a brief (<1 h) alkali bath in 0.1 N NaOH at room temperature to remove humates. The resulting pseudomorph was rinsed to neutrality in multiple changes of NanoPure H_2_O and then gelatinized for 12 h at 60 °C in 0.01 N HCl. The resulting gelatin solution was pipetted into pre-cleaned ultrafilters (retaining > 30 kDa molecular weight gelatin) and centrifuged 3 times for 30 min. and diluted with Nanopure H2O and centrifuged 3 more times for 30 min to desalt the solution (ultrafilter cleaning methods are described in McClure et al. 2011:28–29). Ultrafiltered collagen was lyophilized and weighed to determine percent yield as a first evaluation of the degree of bone collagen preservation.

**Carbon and Nitrogen Measurements**

Carbon and nitrogen measurements of bone collagen were conducted at three labs. Carbon and nitrogen concentrations and stable isotope ratios for Baking Pot (*n* = 10), Barton Creek (*n* = 1), Cahal Pech (*n* = 15), Caracol (*n* = 15), Lower Dover (*n* = 2), Peligroso (*n* = 1), Pook’s Hill (*n* = 6), Ramonal (*n* = 1), and Tipu (*n* = 22), and Xunantunich (*n* = 1; Sample ID XUN05) were measured at the Yale Analytical and Stable Isotope Center (YASIC). Approximately ~0.7 mg of ultrafiltered collagen was combusted in a Costech ECS 4010 Elemental Analyzer and analyzed by a Thermo DeltaPlus XP isotope ratio mass spectrometer (IRMS) connected via a Conflo III interface. The standard uncertainty (Szpak et al. 2017) was ±0.44 ‰ for *δ*^13^C and ±0.40 ‰ for *δ*^15^N.

Carbon and nitrogen isotope analysis of the Caledonia (*n* = 22) and Pacbitun (*n* = 11) materials were performed by the CREAIT Stable Isotope Laboratory (SIL) at the Memorial University of Newfoundland (MUN). One milligram aliquots, weighed into tin capsules, were analyzed on Delta V-Plus I IRMS coupled via continuous flow to a Carlo Erba NA 1500 Series II elemental analyzer (EA) with a standard uncertainty (Szpak et al. 2017) of ±0.2 ‰ for *δ*^13^C and ±0.24 ‰ for *δ*^15^N. The carbon and nitrogen isotopes of 1 mg collagen samples from Xunantunich (*n* = 6) and San Lorenzo (*n* = 1) weighed into tin capsules were analyzed on a Delta Advantage IRMS coupled to a Vario EL Cube EA via continuous flow in the Ján Veizer SIL at the University of Ottawa, with a standard uncertainty (Szpak et al. 2017) of ±0.11 ‰ for *δ*^13^C and ±0.08 ‰ for *δ*^15^N.

Sample quality for all samples was evaluated by % crude gelatin yield, %C, %N, and C:N ratios. C:N ratios for all samples except MARC2571 (Pacbitun, Lot 390) fall between 3.10 and 3.50, indicating good collagen preservation (DeNiro 1985; van Klinken 1999). Because of a higher than acceptable C:N ratio for MARC2571 (3.70), it was removed from subsequent statistical analyses.

Five samples had %C greater than 50 % (expected %C is 40-45 % for archaeological samples). Quality assurance and normalization data for from standards in each run, which were all analyzed at YASIC, fall within the acceptable range accounting for measurement error. The C:N ratio for theses samples also falls within the acceptable ranges. Therefore, we attribute the higher-than-expected C% data to the sample being larger than the ostensible submitted mass. In other words, we believe the issue stems from the initial weighing of samples prior to analysis, likely due to static or vibrations impacting the balance.

**Sulfur Measurements**

Sulfur isotope ratios for Baking Pot (*n* = 10), Barton Creek (*n* = 1), Cahal Pech (*n* = 15), Caracol (*n* = 15), Lower Dover (*n* = 2), Peligroso (*n* = 1), Pook’s Hill (*n* = 6), Ramonal (*n* = 1), Tipu (*n* = 22), and Xunantunich (*n* = 1; Sample ID XUN05) were measured at the Stable Isotope Ratio Facility for Environmental Research at the University of Utah. Carbon and nitrogen measurements for these samples was completed at YASIC, though only data from Cahal Pech (Ebert et al. 2019) and Baking Pot (Hoggarth et al. 2014) is previously reported. For sulfur analysis, approximately 15 mg of collagen for each sample was placed in a tin capsule and analyzed on a Finnigan Delta Plus XL connected with Costech EA 4010 via Finnigan Conflo III.

Sulfur isotope analysis of 15 samples from Caledonia was conducted in the MUN SIL lab. Approximately 10-15 mg for each sample was weighed into tin capsules with 1 mg V_2_O_5_ and analyzed on a MAT 252 IRMS coupled to a Carol Erba NA 1500 Series II EA via continuous flow with a standard uncertainty of ±1.24 ‰. The sulfur isotopes of the remaining three samples from Caledonia, as well as those from Pacbitun were analyzed by the Stable Isotope Laboratory table

at the University of Tennessee Knoxville, where 5 mg samples of collagen were weighed into tin capsules with 1 mg of V_2_O_5_ and analyzed on a Delta V Plus IRMS coupled to a EC S4010 EA. Standard uncertainty and analytical accuracy could not be calculated because check standards were not included in the analysis (Szpak et al. 2017), although analytical precision was ±1.00 ‰.

Finally, samples from Xunantunich (*n*=6) and San Lorenzo (*n*=1) weighing between 20-50 mg were mixed with at least twice their mass of sucrose in tin capsules and analyzed on a Delta Plus XP IRMS coupled to an Isotope Cube EA via continuous flow in the Ján Veizer SIL at the University of Ottawa. Analytical uncertainty was ±0.32 ‰, although this did not include sample duplicates due to the large amount of sample required for analysis.

Sample integrity was assessed using the wt. %S, as well as the C:S and N:S ratios (Nehlich and Richards 2009). Samples with sulfur concentrations between 0.15 % and 0.35 %, C:S values between 300 and 900, and N:S values between 100 and 300 were considered adequately preserved for analysis (Nehlich and Richards 2009).

**Analytical Uncertainty of the Isotopic Measurements**

To compare isotopic data generated by different laboratories under differing conditions it is necessary to report analytical uncertainty to determine whether differences in the isotopic values are meaningfully interpretable (i.e., above that of analytical uncertainty) or the result of analytical or human error. The accepted values of the calibration and check standards [S2-1 Table] were compared to the repeated measurements of these standards during analytical sessions [S2-2 Table] and combined with the repeated measurements of sample replicates, when available [S2-3 Table], to calculated analytical uncertainty following the method outlined by Szpak and colleagues (2017). The analytical uncertainty for each isotope system by laboratory is reported in S2-4 Table.

**Table S2-1. Accepted values of the calibration and check standards for each isotope system by laboratory.**

| **Lab** | **Standard Type** | **Standard Name** | **Material** | **Accepted *δ*^13^C**  **(‰, VPDB)** | **Accepted *δ*^15^N**  **(‰, Atm N_2_)** | **Accepted *δ*^34^S**  **(‰, VCDT)** |
| --- | --- | --- | --- | --- | --- | --- |
| MUN | Calibration | EDTA #2 | EDTA | -40.38±0.01 | -0.83±0.04 |  |
| MUN | Calibration | USGS-62 | Caffeine | -14.79±0.04 | +20.17±0.06 |  |
| MUN | Calibration | IAEA-N-2 | Ammonium Sulfate |  | +20.32±0.09 |  |
| MUN | Check | G-9 | L-glutamic acid | -26.74±0.06 | -2.77±0.18 |  |
| MUN | Check | G-32 | Sulfanilamide | -28.96±0.22 | -3.62±0.25 |  |
| MUN | Check | G-40 | B2155 (protein) | -27.03±0.13 | +5.97±0.08 |  |
| MUN | Calibration | IAEA-S-1 | Silver sulfide |  |  | -0.3 |
| MUN | Calibration | IAEA-S-2 | Silver sulfide |  |  | +22.67±0.15 |
| MUN | Check | NBS-127 | Barium sulfate |  |  | +21.1±0.36 |
| MUN | Check | G-50 | B2155 (casein) |  |  | +6.57±0.8 |
| Ottawa* | Check | C-55 | Glutamic acid | -28.53 | -3.98 |  |
| Ottawa* | Check | S-6 | AG-2 |  |  | -0.71 |
| Ottawa* | Check | S-13131 | Egg |  |  | +3.51 |
| Ottawa* | Check | S-13132 | DCO Liver |  |  | +4.66 |
| Ottawa* | Check | S-13133 | Nova Egg |  |  | +17.59 |
| Yale | Calibration | CN2 | Unknown | -28.3 | -4.5 |  |
| Yale | Calibration | YGA | Unknown | +31.0 | +31.8 |  |
| Yale | Check | Trout | Protein | -29.1±0.2 | +15.7±0.2 |  |
| Yale | Calibration | NZ1 | Unknown |  |  | -0.30 |
| Yale | Calibration | NZ2 | Unknown |  |  | +22.70 |
| Yale | Check | SS | Unknown |  |  | +8.3±0.6 |
| Tennessee** | Calibration | NBS-127 | Barium Sulfate |  |  | +20.30±0.40 |
| Tennessee** | Calibration | IAEA-SO-6 | Barium Sulfate |  |  | -34.10±0.20 |
| Utah | Calibration | PLRM-1 |  |  |  | +17.94 |
| Utah | Calibration | PLRM-2 |  |  |  | -31.94 |
| Utah | Check | SLRM |  |  |  | +16.9±0.50 |

*The laboratory did not provide information regarding the repeated measurements of calibration standards.

**Check standards were not included in the analyses.

**Table S2-2. Mean and standard deviations of the calibration and check standards analyzed during each analytical session by laboratory.**

| **Lab** | **Standard** | **Session** | ***δ*^13^C**  **(‰, VPDB)** | ***n*** | ***δ*^15^N**  **(‰, Atm N_2_)** | ***n*** | ***δ*^34^S**  **(‰, VCDT)** | ***n*** |
| --- | --- | --- | --- | --- | --- | --- | --- | --- |
| MUN | EDTA#2 | 1 | -40.38±0.02 | 6 | -0.83±0.04 | 6 |  |  |
| MUN | EDTA#2 | 2 | -40.38±0.02 | 6 | -0.83±0.06 | 6 |  |  |
| MUN | EDTA#2 | 3 | -40.38±0.1 | 6 | -0.83±0.07 | 6 |  |  |
| MUN | EDTA#2 | 4 | -40.38±0.16 | 6 | -0.83±0.09 | 6 |  |  |
| MUN | EDTA#2 | 5 | -40.38±0.19 | 5 | -0.83±0.05 | 5 |  |  |
| MUN | EDTA#2 | 6 | -40.38±0.16 | 6 | -0.83±0.03 | 6 |  |  |
| MUN | G-40 | 1 | -27.25±0.03 | 4 | +5.93±0.09 | 4 |  |  |
| MUN | G-40 | 2 | -27.24±0.04 | 4 | +5.90±0.10 | 4 |  |  |
| MUN | G-40 | 3 | -27.29±0.04 | 4 | +6.00±0.09 | 4 |  |  |
| MUN | G-40 | 4 | -27.27±0.22 | 4 | +5.95±0.08 | 4 |  |  |
| MUN | G-40 | 5 | -27.34±0.16 | 3 | +5.98±0.11 | 3 |  |  |
| MUN | G-40 | 6 | -27.24±0.18 | 6 | +6.09±0.08 | 6 |  |  |
| MUN | G-9 | 1 | -26.64±0.04 | 12 | -2.64±0.13 | 12 |  |  |
| MUN | G-9 | 2 | -26.71±0.16 | 9 | -2.67±0.14 | 10 |  |  |
| MUN | G-9 | 3 | -26.81±0.11 | 11 | -2.60±0.07 | 10 |  |  |
| MUN | G-9 | 4 | -26.88±0.28 | 11 | -2.58±0.14 | 13 |  |  |
| MUN | G-9 | 5 | -26.75±0.19 | 7 | -2.76±0.12 | 7 |  |  |
| MUN | G-9 | 6 | -26.74±0.15 | 7 | -2.43±0.12 | 7 |  |  |
| MUN | USGS-62 | 1 | -14.79±0.04 | 6 | +20.17±0.07 | 6 |  |  |
| MUN | USGS-62 | 2 | -14.79±0.06 | 6 | +20.17±0.06 | 6 |  |  |
| MUN | USGS-62 | 3 | -14.79±0.11 | 6 | +20.17±0.04 | 6 |  |  |
| MUN | USGS-62 | 4 | -14.79±0.18 | 6 | +20.17±0.06 | 6 |  |  |
| MUN | USGS-62 | 5 | -14.79±0.2 | 5 | +20.17±0.02 | 5 |  |  |
| MUN | USGS-62 | 6 | -14.79±0.11 | 7 | +20.17±0.12 | 6 |  |  |
| MUN | G-32 | 6 | -28.99±0.16 | 6 | -3.58±0.10 | 6 |  |  |
| MUN | IAEA-N-2 | 6 |  | 0 | +20.3 | 1 |  |  |
| MUN | IAEA-S-1 | 7 |  |  |  |  | -0.30±.30 | 7 |
| MUN | IAEA-S-1 | 8 |  |  |  |  | -0.36±0.27 | 7 |
| MUN | IAEA-S-2 | 7 |  |  |  |  | +22.67±0.08 | 9 |
| MUN | IAEA-S-2 | 8 |  |  |  |  | +22.67±0.22 | 9 |
| MUN | NBS-127 | 7 |  |  |  |  | +21.67±1.19 | 4 |
| MUN | NBS-127 | 8 |  |  |  |  | +21.85±0.63 | 4 |
| MUN | G-50 | 7 |  |  |  |  | +7.04±0.06 | 2 |
| MUN | G-50 | 8 |  |  |  |  | +5.99±0.18 | 2 |
| Ottawa | C-55 | Unknown | -28.6±0.04 | 4 | -4.0±0.06 | 4 |  |  |
| Ottawa | S-6 | Unknown |  |  |  |  | -0.72±0.13 | 5 |
| Ottawa | S-13131 | Unknown |  |  |  |  | +2.95 | 1 |
| Ottawa | S-13132 | Unknown |  |  |  |  | +4.57 | 1 |
| Ottawa | S-13133 | Unknown |  |  |  |  | +17.63 | 1 |
| YASIC | CN2 | 1 | -28.32±0.14 | 5 | -4.52±0.06 | 5 |  |  |
| YASIC | CN2 | 2 | -28.32±0.07 | 3 | -4.52±0.21 | 3 |  |  |
| YASIC | CN2 | 3 | -28.32±0.20 | 4 | -4.52±0.24 | 4 |  |  |
| YASIC | CN2 | 4 | -28.32±0.03 | 3 | -4.52±0.08 | 3 |  |  |
| YASIC | YGA | 1 | +31.02±0.13 | 4 | +31.80±0.04 | 4 |  |  |
| YASIC | YGA | 2 | +31.02±0.12 | 3 | +31.80±0.21 | 3 |  |  |
| YASIC | YGA | 3 | +31.02±0.23 | 4 | +31.80±0.17 | 4 |  |  |
| YASIC | YGA | 4 | +31.02±0.11 | 3 | +31.80±0.06 | 3 |  |  |
| YASIC | Trout | 1 | -29.17±0.14 | 4 | +15.72±0.05 | 4 |  |  |
| YASIC | Trout | 2 | -28.97±0.06 | 3 | +15.53±0.22 | 3 |  |  |
| YASIC | Trout | 3 | -29.24±0.06 | 3 | +15.56±0.17 | 3 |  |  |
| YASIC | Trout | 4 | -29.12±0.08 | 4 | +15.53±0.10 | 4 |  |  |
| YASIC | NZ1 | 1 |  |  |  |  | +0.74±0.81 | 2 |
| YASIC | NZ1 | 2 |  |  |  |  | +1.16±1.65 | 3 |
| YASIC | NZ1 | 3 |  |  |  |  | -0.29±0.60 | 3 |
| YASIC | NZ1 | 4 |  |  |  |  | -0.23±0.87 | 3 |
| YASIC | NZ2 | 1 |  |  |  |  | +22.59±0.16 | 3 |
| YASIC | NZ2 | 2 |  |  |  |  | +22.33±1.13 | 3 |
| YASIC | NZ2 | 3 |  |  |  |  | +22.67±0.36 | 3 |
| YASIC | NZ2 | 4 |  |  |  |  | +22.61±1.31 | 3 |
| YASIC | SS | 1 |  |  |  |  | +8.38±0.41 | 3 |
| YASIC | SS | 2 |  |  |  |  | +7.79±1.57 | 4 |
| YASIC | SS | 3 |  |  |  |  | +8.08±1.05 | 4 |
| YASIC | SS | 4 |  |  |  |  | +8.19±0.22 | 3 |
| Tennessee | IAEA-SO-2 | 1 |  |  |  |  | -34.05±0.06 | 3 |
| Tennessee | IAEA-SO-2 | 2 |  |  |  |  | -34.05±1.5 | 8 |
| Tennessee | IAEA-SO-2 | 3 |  |  |  |  | -33.89±0.52 | 6 |
| Tennessee | IAEA-SO-2 | 4 |  |  |  |  | -34.05±0.41 | 6 |
| Tennessee | IAEA-SO-2 | 5 |  |  |  |  | -34.05±0.99 | 6 |
| Tennessee | IAEA-SO-2 | 6 |  |  |  |  | -34.05±0.80 | 8 |
| Tennessee | IAEA-SO-2 | 7 |  |  |  |  | -33.75±0.93 | 7 |
| Tennessee | NBS-127 | 1 |  |  |  |  | +21.12±0.12 | 2 |
| Tennessee | NBS-127 | 2 |  |  |  |  | +21.15±0.26 | 7 |
| Tennessee | NBS-127 | 3 |  |  |  |  | +21.12±0.33 | 6 |
| Tennessee | NBS-127 | 4 |  |  |  |  | +21.12±0.26 | 5 |
| Tennessee | NBS-127 | 5 |  |  |  |  | +21.12±0.35 | 5 |
| Tennessee | NBS-127 | 6 |  |  |  |  | +21.12±0.65 | 8 |
| Tennessee | NBS-127 | 7 |  |  |  |  | +21.12±0.40 | 6 |
| Utah | PLRM-1 | 1 |  |  |  |  | +17.94±0.25 | 3 |
| Utah | PLRM-1 | 2 |  |  |  |  | +17.94±0.26 | 3 |
| Utah | PLRM-1 | 3 |  |  |  |  | +17.94±0.22 | 3 |
| Utah | PLRM-2 | 1 |  |  |  |  | -31.94±0.22 | 3 |
| Utah | PLRM-2 | 2 |  |  |  |  | -31.94±0.37 | 3 |
| Utah | PLRM-2 | 3 |  |  |  |  | -31.94±0.05 | 3 |
| Utah | SLRM | 1 |  |  |  |  | +16.96±0.04 | 3 |
| Utah | SLRM | 2 |  |  |  |  | +16.81±0.26 | 3 |
| Utah | SLRM | 3 |  |  |  |  | +16.79±0.09 | 3 |

**Table S2-3. Stable carbon, nitrogen, and sulfur isotope values of sample replicates analyzed during each analytical session by laboratory.**

| **Lab** | **Sample Lab #** | **Session** | ***δ*^13^C**  **(‰, VPDB)** | ***n*** | ***δ*^15^N**  **(‰, Atm N_2_)** | ***n*** | ***δ*^34^S**  **(‰, VCDT)** | ***n*** |
| --- | --- | --- | --- | --- | --- | --- | --- | --- |
| MUN | MARC 4267 | 1 | -12.70±0.02 | 3 | +9.59±0.17 | 3 |  |  |
| MUN | MARC 4302 | 2 | -20.35 ±0.05 | 3 | +6.40±0.04 | 3 |  |  |
| MUN | MARC 4161 | 3 | -22.18±0.01 | 3 | +4.41±0.05 | 3 |  |  |
| MUN | MARC 4186 | 4 | -22.02±0.03 | 3 | +4.02±0.09 | 3 |  |  |
| MUN | MARC 4331 | 5 | -21.35±0.05 | 3 | +4.59±0.14 | 3 |  |  |
| MUN | MARC 4497 | 6 | -14.16±0.23 | 3 | +10.04±0.18 | 3 |  |  |
| MUN* | MARC 2514 | 7 & 8 |  |  |  |  | +9.64±0.90 | 2 |
| MUN* | MARC 2515 | 7 & 8 |  |  |  |  | +13.01±0.82 | 2 |
| MUN* | MARC 2516 | 7 & 8 |  |  |  |  | +11.52±1.94 | 2 |
| MUN* | MARC 2517 | 7 & 8 |  |  |  |  | +12.93±1.70 | 2 |
| MUN* | MARC 2518 | 7 & 8 |  |  |  |  | +11.63±0.88 | 2 |
| MUN* | MARC 2519 | 7 & 8 |  |  |  |  | +10.21±0.76 | 2 |
| MUN* | MARC 2521 | 7 & 8 |  |  |  |  | +12.01±1.30 | 2 |
| MUN* | MARC 2523 | 7 & 8 |  |  |  |  | +12.68±0.02 | 2 |
| MUN* | MARC 2528 | 7 & 8 |  |  |  |  | +13.73±0.00 | 2 |
| MUN | MARC 2532 | 8 |  |  |  |  | +13.67±0.76 | 2 |
| Ottawa | MARC 2519B | Unknown | -11.77±0.04 | 2 | +11.62±0.05 | 2 |  |  |
| Ottawa | MARC 2523B | Unknown | -8.46±0.11 | 2 | +10.52±0.06 | 2 |  |  |
| Ottawa | MARC 2532B | Unknown | -8.64±0.03 | 2 | +9.22±0.03 | 2 |  |  |
| Ottawa | MARC 4406 | Unknown | -7.86±0.01 | 2 | +9.20±0.05 | 2 |  |  |
| Ottawa | MARC 4564 | Unknown | -20.48±0.00 | 2 | +3.9±0.03 | 2 |  |  |
| Ottawa | MARC 4574 | Unknown | -9.19±0.07 | 2 | +10.12±0.01 | 2 |  |  |
| Ottawa | MARC 4585 | Unknown | -21.42±0.02 | 2 | +4.7±0.01 | 2 |  |  |
| Ottawa | MARC 4592 | Unknown | -9.48±0.03 | 2 | +8.78±0.02 | 2 |  |  |
| Ottawa | MARC 4757 | Unknown | -9.63±0.03 | 2 | +9.89±0.09 | 2 |  |  |
| Ottawa | MARC 4924 | Unknown | -10.66±0.29 | 2 | +8.17±0.11 | 2 |  |  |
| YASIC | CHP 15 | Unknown | -8.15±0.50 | 2 | +9.75±0.21 | 2 |  |  |
| YASIC | TLK 10 | Unknown | -13.5±1.27 | 2 | +8.7±0.28 | 2 |  |  |
| YASIC | ZBN 1 | Unknown | -8.65±0.35 | 2 | +9.2±1.13 | 2 |  |  |
| YASIC | ZBN 13 | Unknown | -11.8±0.14 | 2 | +8.55±0.21 | 2 |  |  |
| Tennessee* | MARC 2558 | 6 & 7 |  |  |  |  | +11.45±0.52 | 2 |
| Tennessee* | MARC 2561 | 6 & 7 |  |  |  |  | +13.30±0.18 | 2 |
| Tennessee* | MARC 3451 | 6 & 7 |  |  |  |  | +15.46±1.77 | 2 |
| Tennessee* | MARC 3458 | 6 & 7 |  |  |  |  | +13.26±0.06 | 2 |
| Tennessee* | MARC 4155 | 6 & 7 |  |  |  |  | +14.17±0.70 | 2 |
| Tennessee | MARC 4161 | 7 |  |  |  |  | +8.61±0.54 | 2 |
| Tennessee | MARC 4192 | 7 |  |  |  |  | +4.56±0.18 | 2 |
| Tennessee* | MARC 4255 | 1 & 7 |  |  |  |  | +12.48±0.81 | 2 |
| Tennessee* | MARC 4260 | 1 & 2 |  |  |  |  | +12.4±1.40 | 2 |
| Tennessee* | MARC 4261 | 1 & 2 |  |  |  |  | +9.53±0.12 | 2 |
| Tennessee* | MARC 4262 | 1, 2, & 7 |  |  |  |  | +12.99±0.06 | 3 |
| Tennessee* | MARC 4263 | 1 & 2 |  |  |  |  | +12.81±0.16 | 2 |
| Tennessee* | MARC 4267 | 1 & 2 |  |  |  |  | +8.52±0.41 | 2 |
| Tennessee* | MARC 4268 | 1 & 2 |  |  |  |  | +10.74±0.28 | 2 |
| Tennessee* | MARC 4270 | 1, 2, & 7 |  |  |  |  | +12.06±0.38 | 3 |
| Tennessee* | MARC 4271 | 1 & 2 |  |  |  |  | +14.02±0.32 | 2 |
| Tennessee* | MARC 4272 | 1 & 2 |  |  |  |  | +12.22±2.13 | 2 |
| Tennessee* | MARC 4273 | 1 & 2 |  |  |  |  | +12.34±0.46 | 2 |
| Tennessee* | MARC 4274 | 1 & 2 |  |  |  |  | +17.13±0.01 | 2 |
| Tennessee* | MARC 4294 | 3 & 7 |  |  |  |  | +13.73±0.64 | 2 |
| Tennessee* | MARC 4303 | 3 & 7 |  |  |  |  | +13.83±0.42 | 2 |
| Tennessee* | MARC 4305 | 3 & 7 |  |  |  |  | +8.08±1.85 | 2 |
| Tennessee* | MARC 4320 | 2 & 7 |  |  |  |  | +12.72±0.97 | 2 |
| Tennessee* | MARC 4328 | 2 & 7 |  |  |  |  | +13.94±0.66 | 2 |
| Tennessee* | MARC 4332 | 4 & 7 |  |  |  |  | +13.54±0.68 | 2 |
| Tennessee* | MARC 4406 | 4 & 7 |  |  |  |  | +11.59±0.23 | 2 |
| Tennessee* | MARC 4522 | 4 & 7 |  |  |  |  | -1.26±1.53 | 2 |
| Tennessee* | MARC 4533 | 4 & 7 |  |  |  |  | +12.64±0.71 | 2 |
| Tennessee* | MARC 4553 | 5 & 7 |  |  |  |  | -2.81±2.35 | 2 |
| Tennessee* | MARC 4555 | 5 & 7 |  |  |  |  | +12.26±1.39 | 2 |

*Sample replicates analyzed during different analytical sessions.

Note: The *δ*^34^S values of sample replicates were not analyzed at the Ján Veizer Laboratory in Ottawa, YASIC, or Utah.

**Table S2-4.** **Analytical uncertainty of each isotope system by laboratory.**

| **Isotope System** | **Laboratory** | **Measurement Precision** | | | **Precision Specific to the Samples** | **Analytical Precision** | **Analytical Accuracy** | **Standard Uncertainty** |
| --- | --- | --- | --- | --- | --- | --- | --- | --- |
|  |  | **Calibration Standards** | **Check Standards** | **Calibration and Check Standards** |  |  |  |  |
| ***δ*^13^C**  **(‰, VPDB)** | MUN | ±0.13 | ±0.12 | ±0.15 | ±0.10 | ±0.17 | ±0.23 | ±0.28 |
|  | Ottawa | N/A | ±0.04 | ±0.04 | ±0.10 | ±0.08 | ±0.07 | ±0.11 |
|  | YASIC | ±0.15 | ±0.10 | ±0.14 | ±0.50 | ±0.38 | ±0.22 | ±0.44 |
| ***δ*^15^N**  **(‰, Atm N_2_)** | MUN | ±0.06 | ±0.12 | ±0.10 | ±0.12 | ±0.13 | ±0.20 | ±0.24 |
|  | Ottawa | N/A | ±0.06 | ±0.06 | ±0.06 | ±0.07 | ±0.02 | ±0.08 |
|  | YASIC | ±0.15 | ±0.14 | ±0.15 | ±0.43 | ±0.33 | ±0.22 | ±0.40 |
| ***δ*^34^S**  **(‰, VCDT)** | MUN | ±0.25 | ±0.83 | ±0.45 | ±1.08 | ±0.89 | ±0.86 | ±1.24 |
|  | Tennessee* | ±0.74 | N/A | ±0.74 | ±0.96 | ±1.00 | N/A | N/A |
|  | Ottawa | ±0.20** | ±0.13 | ±0.13 | N/A | ±0.13 | ±0.29 | ±0.32 |
|  | YASIC | ±0.99 | ±1.24 | ±1.01 | N/A | ±1.01 | ±0.67 | ±1.21 |
|  | Utah | ±0.26 | ±0.18 | ±0.23 | N/A | ±0.23 | ±0.51 | ±0.56 |

*Analytical accuracy, and therefore analytical uncertainty, could not be calculated because check standards were not included in analytical sessions.

**Value provided by the lab but not included in calculation of analytical uncertainty.

**Previous Sulfur Measurements from the Sample and Inter-Lab Variation**

A recent study by Green (2016) examined the diet and movement of individuals from Cahal Pech, which included the analysis of sulfur isotopes from five individuals [S2-5 Table]. These samples were analyzed at the UC-Davis Stable Isotope Facility and were measured using an Elementar Vario ISOTOPE cube interfaced to a SerCon 20-22 IRMS (Green 2016:137). Results showed no correlation between *δ*^34^S and *δ*^15^N values, which could potentially distinguish between marine and terrestrial diets (Green 2016:75-76). Instead, similar results between four individuals, all of whom were determined to be local based on associated strontium isotope values, suggesting the utility of sulfur isotope data as an indicator of origin (in this case at death). Our *δ*^34^S values for these individuals are approximately +2.0 ‰ relative to the previously reported results, which falls within the maximum analytical uncertainty reported for *δ*^34^S values in this study (±1.24 ‰; Table S8) but beyond the expected range of variation for an individual (±0.6 ‰; Nehlich and Richards 2009:61). This is likely due to inter-laboratory variability in analysis (Rand 2021) and differences in sample preparation methods with and without ultrafiltration (Privat et al. 2007). An outlier individual (Plaza G, Burial 1) has been documented as non-local to the Belize Valley by Awe and colleagues (2017) and has “non-local” *δ*^34^S values in both studies, although the studies obtained extremely divergent values.

**Table S2-5.** **Comparison of previously reported sulfur isotope results from Green (2016:91, Table 6.2) to reanalysis of samples performed for this study.**

| **Provenience** | **Green 2016** | | | | **This study** | | | |
| --- | --- | --- | --- | --- | --- | --- | --- | --- |
|  | ***δ*^34^S_VCDT_ (‰)** | **Wt% S** | **C:S** | **N:S** | ***δ*^34^S_VCDT_ (‰)** | **Wt% S** | **C:S** | **N:S** |
| Cahal Pech Str. B4 (1 sub) | +11.71 | 0.3 | 342.5 | 103.9 | +13.3 | 0.21 | 642.1 | 196.2 |
| Cahal Pech Str. B4 Level 5 Burial 1 | +12.56 | 0.2 | 554.9 | 173.0 | +13.6 | 0.20 | 632.9 | 197.0 |
| Cahal Pech Plaza B EU-13 Burial 1 | +11.77 | 0.2 | 526.5 | 161.5 | +13.2 | 0.22 | 551.7 | 168.8 |
| Cahal Pech Str. C2 Burial 1 | +11.54 | 0.2 | 524.1 | 162.3 | +13.5 | 0.22 | 596.9 | 181.2 |
| Cahal Pech Plaza G Burial 1 | +6.89 | 0.3 | 347.1 | 107.5 | +16.7 | 0.18 | 669.1 | 197.7 |

**References**

Brown TA, Nelson DE, Vogel JS, Southon JS. Extraction by Modified Longin Method. Radiocarbon 1988; 30: 171-177. doi: 10.1017/S0033822200044118.

DeNiro MJ. Post-mortem preservation and alteration of in-vivo bone collagen isotope ratios in relation to paleodietary reconstruction. Nature 1985; 317: 806-809. doi: 10.1038/317806a0.

Ebert CE, Hoggarth JA, Awe JA, Culleton BJ, Kennett DJ. The Role of Diet in Resilience and Vulnerability to Climate Change among Early Agricultural Communities in the Maya Lowlands. Curr. Anthropol. 2019; 60(4): 589-601. doi: 10.1086/704530.

Green KA. The use of stable isotope analysis on burials at Cahal Pech, Belize in order to identify trends in mortuary practices over time and space. Ph.D. Dissertation, University of Montana. 2016. Available from: <https://scholarworks.umt.edu/cgi/viewcontent.cgi?article=11962&context=etd>.

Hoggarth JA, Culleton BJ, Awe JJ, Kennett DJ. Questioning Postclassic Continuity at Baking Pot, Belize Using Direct AMC ^14^C Dating of Human Burials. Radiocarbon 2014; 56(3): 1057-1075. doi: 10.2458/56.18100.

Longin R. New Method of Collagen Extraction for Radiocarbon Dating. Nature 1971; 230: 241-424. doi: 10.1038/230241a0.

McClure SB, Garcia Puchol O, Culleton BJ. AMS Dating of Human Bone from Cova de la Pastora: New Evidence of Ritual Continuity in the Prehistory of Eastern Spain. Radiocarbon 2011; 52: 25-32. doi: OI: 10.1017/S0033822200045008.

Nehlich O, Richards MP. Establishing collagen quality criteria for sulphur isotope analysis of archaeological bone collagen. Archaeol. Anthropol. Sci. 2009; 1: 59-75. doi: 10.1007/s12520-009-0003-6.

Privat KL, O’Connell TC, Hedges REM. The distinction between freshwater- and terrestrial-based diets: Methodological concerns and archaeological applications of sulphur stable isotope analysis. J. Archaeol. Sci. 2007; 34: 1197-1204. doi: 10.1016/j.jas.2006.10.008

Rand AJ. Prehispanic and Colonial Maya subsistence and migration: Contributions from stable sulphur isotope analysis [dissertation]. St. John’s (ON): Memorial University of Newfoundland; 2021.

Szpak P, Metcalfe J, Macdonald RA. Best practices for calibrating and reporting stable isotope measurements in archaeology. J. Archaeol. Sci. Rep. 2017; 13:609-616. doi: 10.1016/j.jasrep.2017.05.007.

van Klinken GJ. Bone Collagen Quality Indicators for Palaeodietary and Radiocarbon Measurements. J. Archaeol. Sci. 1999; 26: 687-695. doi: 10.1006/jasc.1998.0385.
