# Supplementary material for: Sulfur isotopes as a proxy for human diet and mobility from the preclassic through colonial periods in the Eastern Maya lowlands
Source: PLoS One. 2021 Aug 12;16(8):e0254992. doi: 10.1371/journal.pone.0254992 (PMC8360522; doi:10.1371/journal.pone.0254992)
Supplement: S3 Text — (DOCX) [file pone.0254992.s005.docx]

**S3 Text: Statistical Analyses of *δ*^13^C, *δ*^15^N, and *δ*^34^S Data**

**Single Factor Statistical Analyses**

Single factor analyses for each category of isotopic data (*δ*^13^C, *δ*^15^N, and *δ*^34^S) are described below for samples that passed quality control measures. Isotopic data were evaluated based on associated geographic zone, associated time period, and age and sex of individuals when that information was available. Specific statistical tests chosen are described below where appropriate. We did not include samples that failed to meet quality control criteria (see Methods in main text).

**Geographic Zone**

To investigate the relationship between diet and geographic location, samples were assigned to geographic zones according to the discussion in the manuscript. Summary statistics for *δ*^13^C, *δ*^15^N, and *δ*^34^S values by geographic zone are presented in Table S3-1. ANOVA (analysis of variance) was used to evaluate differences between mean *δ*^13^C, *δ*^15^N, and *δ*^34^S values between geographic zones (Drennan 2009:169). This was complemented with Kruskal-Wallis H test since the distributions of many groups strongly deviate from normal and variances differ because of the presence of outliers in the datasets (Gauthier and Hawley 2015). Significance was set at α = 0.05 for all tests.

There were no statistical differences between geographic zones for *δ*^13^C values (*n* = 99; ANOVA, *df* = 2, *F* = 2.24, *p* = .1122; Kuskal-Wallis, *H* = 4.599, *p* = .102) or *δ*^15^N values (*n* = 99; ANOVA, *df* = 2, *F* = .4217, *p* = .652; Kuskal-Wallis, *H* = .2135, *p* = .899). Though we could not include the Maya Mountains in these calculations because of the small sample size (*n* = 2), the *δ*^13^C and *δ*^15^N values for these sites falls well within the ranges for other geographic zones. Sulfur data does, however, vary significantly between geographic zones (*n* = 100; ANOVA, *df* = 2, *F* = 13.55, *p* = < .00001; Kuskal-Wallis, *H* = 27.13, *p* = < .00001). These differences are explored in more detail below in Section 4.

**Table S3-1: Mean *δ*^13^C, *δ*^15^N, and *δ*^34^S values for human bone collagen by Eastern lowland geographic zone.**

| **Eastern Lowland Geographic Zone** | ***n*** | ***δ*^13^C**  **(‰ VPDB)** | **st**  **dev** | ***δ*^15^N**  **(‰ Atm N_2_)** | **st**  **dev** | ***δ*^34^S**  **(‰ VCDT)** | **st**  **dev** |
| --- | --- | --- | --- | --- | --- | --- | --- |
| Belize Valley | 28 | –10.7 | 1.6 | +9.2 | 1.1 | +13.4 | 1.6 |
| Belize Valley Southern Periphery | 38 | –10.1 | 1.4 | +9.0 | 0.7 | +12.2 | 1.4 |
| Vaca Plateau | 33 | –9.9 | 1.6 | +9.2 | 1.2 | +10.7 | 2.9 |
| Maya Mountains | 2 | –10.7 | 0.5 | +9.6 | 1.1 | +8.5 | 0.0 |

**Time Period**

To investigate shifts in isotopic values through time, samples (*n*=105) were assigned temporal categories (see Table 1 in manuscript). Early and Late Postclassic were combined into a single temporal category because of small sample size for the Early Classic and based on similarities in material culture during these two periods in the Eastern lowlands. One sample from Pacbitun (MARC2550) was not included in the temporal comparisons of data described below because no time period could be assigned for this individual, even though the sample met quality control measures. Summary statistics are provided below in Table S3-2.

Data were examined through single pair-wise statistical testing. The *t*-test for independent groups (two parameters) assuming unequal variance was used to determine significant differences between the means within each group (Ruxton 2006). Additionally, the nonparametric Mann–Whitney *U* test (two parameters) for independent samples was used where applicable due to the small sample size since not all groups were normally distributed. Significance was set at α = 0.05 for all tests and the results are presented in Table S3-3.

Comparisons between *δ*^13^C through time show a significant increase of values for three time periods:

1. From the Late Preclassic (*n*=5; *x̅* ± 2*σ* = -12.1 ± 1.0 ‰) to Early Classic period (*n*=7; *x̅* ± 2*σ* = -10.1 ± 0.7 ‰) (*t* = -3.80; *df* = 7; *p* < 0.01)
2. From the Late Classic (*n*=53; *x̅* ± 2*σ* = -10.1 ± 1.6 ‰) to Terminal Classic (*n*=13; *x̅* ± 2*σ* = -11.1 ± 1.7 ‰) (*t* = 2.03; *df* = 18; *p* < 0.05)
3. From the Terminal Classic to the Postclassic (*n*=11; *x̅* ± 2*σ* = -9.6 ± 1.6 ‰) (*t* = -2.37; *df* = 22; *p* < 0.05).

There were no statically identifiable differences between other time periods (Fig 4 in text). There were no significant differences in *δ*^15^N values through time (Figure S3-1). Significant differences are noted in *δ*^34^S values (Figure S3-2). Significant differences are also noted in *δ*^34^S values when they are examined through time.

There was a decrease in *δ*^34^S from the Early (*n*=7; *x̅* ± 2*σ* = +13.1 ± 1.3 ‰) to Late Classic (*n=*54; *x̅* ± 2*σ* = +11.2 ± 2.7 ‰) (*t* = 3.19; *df* = 14; *p* < 0.01) and from the Late to Terminal Classic (*n=*13; *x̅* ± 2*σ* = +12.7 ± 1.3 ‰) (*t* = -2.89; *df* = 41; *p* < 0.01). These shifts are likely influenced by sampling biases instead of dietary change. Belize Valley individuals represent ~70 % of the sample for the Early Classic. In the Late Classic, individuals from the Vaca Plateau account for the largest proportion of the sample (57 %), with the Belize Valley (22 %) and Belize Valley Southern Periphery (16 %) represented by smaller numbers. The Late Classic also includes the only two Maya Mountains individuals in this study from Peligroso and Ramonal, who had some of the lowest *δ*^34^S values in the dataset. The transition from the Late to Terminal Classic is also associated with a shift in geographic zone representation, with no individual from the Vaca Plateau. Therefore, significant changes through time more likely correlate with the predominant geographic zones represented by our sulfur isoscape model in each time period.

**Table S3-2. Mean isotopic values for human bone collagen by time period.**

| **Time Period** | ***n*** | **δ^13^C**  **(‰ VPDB)** | **st**  **dev** | **δ^15^N**  **(‰ Atm N_2_)** | **st**  **dev** | **δ^34^S**  **(‰ VCDT)** | **st**  **dev** |
| --- | --- | --- | --- | --- | --- | --- | --- |
| Late Preclassic | 5 | –12.1 | 1.0 | +8.4 | 0.5 | +13.5 | 1.5 |
| Early Classic | 7 | –10.1 | 0.7 | +8.8 | 1.5 | +13.1 | 1.3 |
| Late Classic | 53 | –10.1 | 1.6 | +9.1 | 1.0 | +11.2 | 2.7 |
| Terminal Classic | 13 | –11.0 | 1.7 | +9.4 | 1.1 | +12.7 | 1.3 |
| Late Postclassic | 11 | –9.6 | 1.6 | +9.2 | 0.7 | +12.5 | 1.6 |
| Colonial | 11 | –9.8 | 0.7 | +9.2 | 0.6 | +13.1 | 1.4 |

**Table S3-3. Results of statistical analyses for isotopic values by time period.** Numbers highlighted in green indicate statistically significant values.

| **Group** | ***n*** | **Variables** | ***t-*test (*p*)** | **Mann-Whitney U (*p*)** |
| --- | --- | --- | --- | --- |
| Late Preclassic vs Early Classic | 5;7 | δ^13^C | **< 0.01** | **< 0.01** |
|  |  | δ^15^N | 0.292 | 0.500 |
|  |  | δ^34^S | 0.317 | 0.436 |
| Early Classic vs Late Classic | 7;53 | δ^13^C | 0.499 | 0.389 |
|  |  | δ^15^N | 0.293 | 0.082 |
|  |  | δ^34^S | **< 0.01** | **< 0.05** |
| Late Classic vs Terminal Classic | 53;13 | δ^13^C | **< 0.05** | **< 0.05** |
|  |  | δ^15^N | 0.129 | 0.125 |
|  |  | δ^34^S | **< 0.01** | **< 0.05** |
| Terminal Classic vs Postclassic | 13;11 | δ^13^C | **< 0.05** | **< 0.05** |
|  |  | δ^15^N | 0.208 | 0.492 |
|  |  | δ^34^S | 0.424 | 0.500 |
| Postclassic vs Colonial | 11;11 | δ^13^C | 0.296 | 0.164 |
|  |  | δ^15^N | 0.457 | 0.460 |
|  |  | δ^34^S | 0.185 | 0.421 |


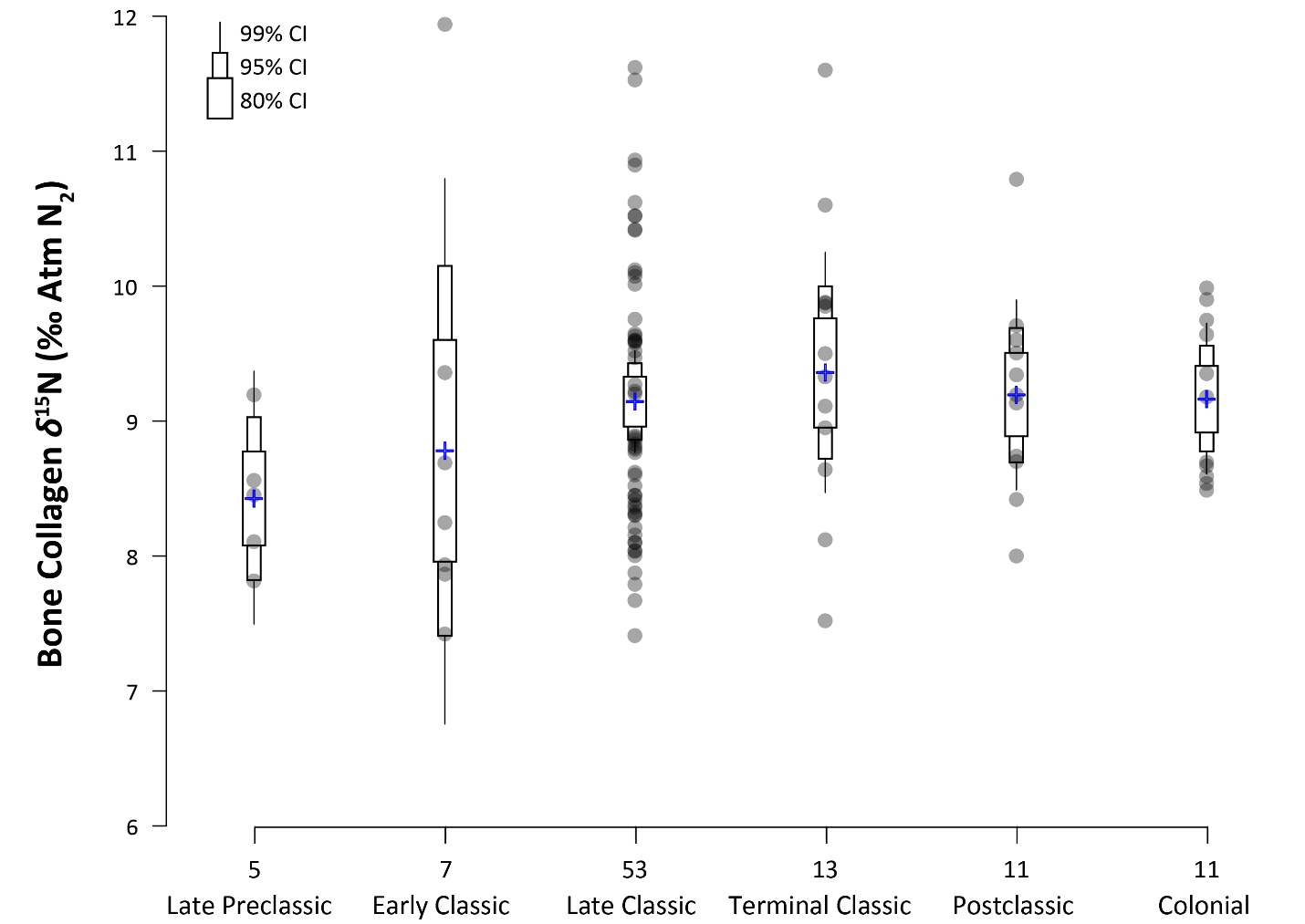


**Figure S3-1. Bullet graph of stable nitrogen (*δ*^15^N) isotope values for human bone collagen.** Data points are shown as circles with the means for each time period indicated by a cross. The bullet graph shows the 80%, 95%, and 99% confidence intervals (CIs; thickest to thinnest bullets) around the mean.


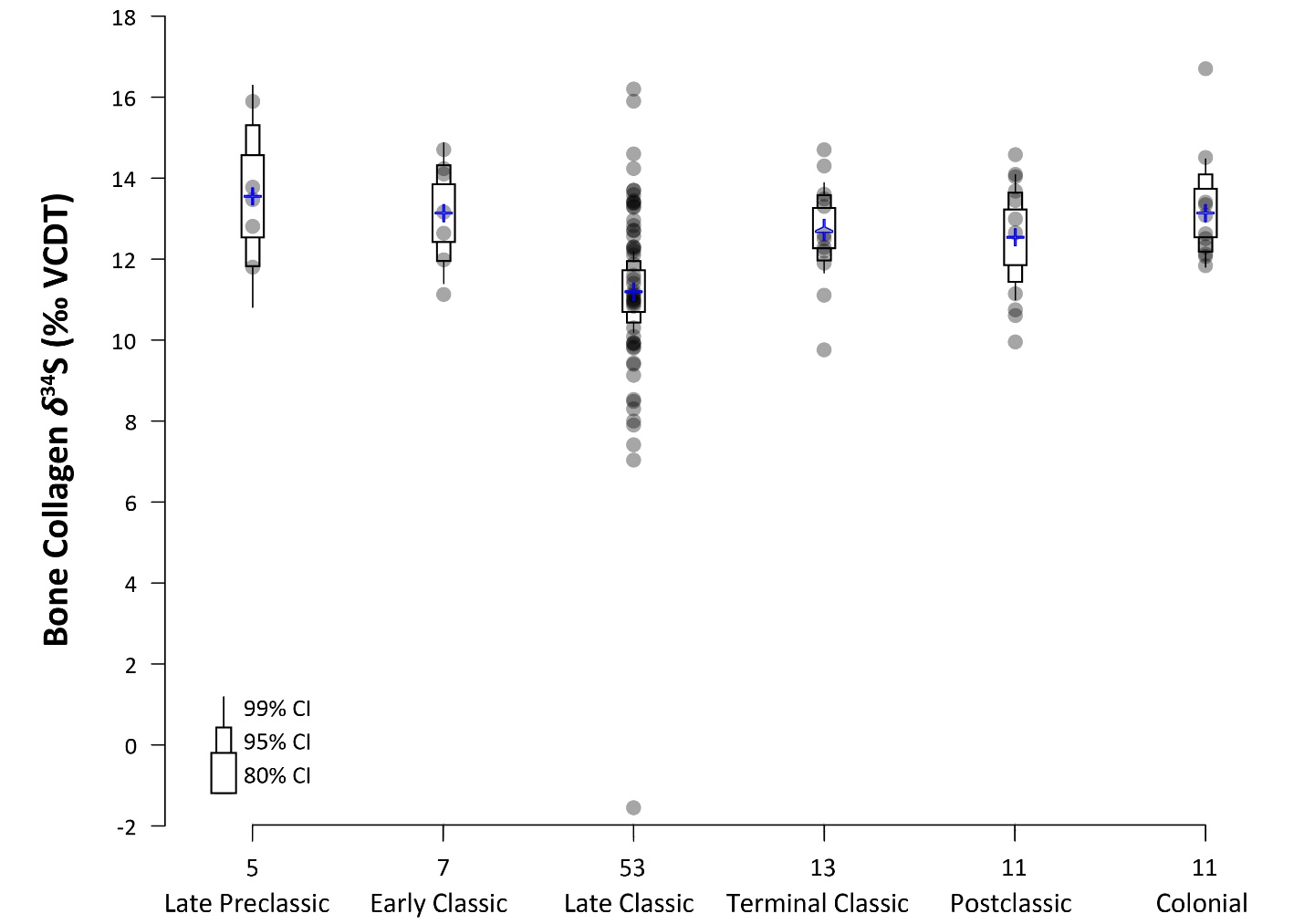


**Figure S3-2. Bullet graph of stable sulfur (*δ*^34^S) isotope values for human bone collagen.** Data points are shown as circles with the means for each time period indicated by a cross. The bullet graph shows the 80%, 95%, and 99% confidence intervals (CIs; thickest to thinnest bullets) around the mean.

**Age and Sex**

We also examined if there was an age or sex effect influencing isotope values. We compared *δ*^13^C, *δ*^15^N, and *δ*^34^S values for all individuals in the sample for which age could be determined (subadults *n*=12, adults *n*=65). Because only four individuals were identified as infants >3-4 yrs, we included them within the sub-adult category. As a result of small sample sizes, or lack of samples in some cases, we could not undertake age-related analyses at the site level or by time period, though we present summary statistics for these data by site in Tables S3-4 and S3-5.

There are no statistical differences between adults and subadults for *δ*^13^C and *δ*^34^S (Table S3-6). In contrast, *δ*^15^N is significantly higher for subadults compared to adults (*t* = -2.03; *df* = 12; *p* = .03). This pattern corresponds with published stable isotopic data for the Maya lowlands that document significantly higher *δ*^15^N for infants (less than 3-4 yrs) related in consumption of breast milk, resulting in a positive trophic shift (Williams et al. 2005; Wright 2013; Wright and Schwarcz 1998; see also Reynard and Tuross 2015).

We also compared *δ*^13^C, *δ*^15^N, and *δ*^34^S values for all individuals in the sample for which biological sex could be determined (female *n*=16, male *n*=25). To be conservative in our interpretations, we did not include data for individuals where sex was designated as probable female (“F?”) or probable male (“M?”). Again, small sample sizes, or lack of samples in some cases, for each site and time period prohibited comparison based on these variables. Summary statistics for these data by site in Tables S3-7 and S3-8. No statistically significant differences were found for *δ*^13^C, *δ*^15^N, and *δ*^34^S values between females and males (Table S3-9).

**Table S3-4.** **Mean** ***δ*^13^C and** ***δ*^15^N values for human bone collagen by age category for sites where these data are available.**

| **Site** | **Adult** | | | | | **Subadult** | | | | |
| --- | --- | --- | --- | --- | --- | --- | --- | --- | --- | --- |
|  | ***n*** | ***δ*^13^C**  **(‰ VPDB)** | **SD** | ***δ*^15^N**  **(‰ Atm N_2_)** | **SD** | ***n*** | ***δ*^13^C**  **(‰ VPDB)** | **SD** | ***δ*^15^N**  **(‰ Atm N_2_)** | **SD** |
| Baking Pot | 1 | –13.4 | -- | +8.1 | -- | 1 | –12.7 | -- | +11.6 | -- |
| Barton Creek |  |  |  |  |  | 1 | –9.0 | -- | +9.3 | -- |
| Cahal Pech | 9 | –10.6 | 1.7 | +8.5 | 0.8 | 4 | –9.1 | 0.9 | +9.8 | 0.9 |
| Caledonia | 17 | –10.0 | 1.9 | +8.8 | 0.9 | 1 | –11.8 | -- | +11.6 | -- |
| Lower Dover | 1 | –9.5 | -- | +8.7 | -- |  |  |  |  |  |
| Pacbitun | 8 | –9.9 | 1.7 | +8.9 | 0.7 | 1 | –13.3 | -- | +7.5 | -- |
| Pook's Hill | 4 | –11.1 | 0.6 | +8.4 | 0.3 |  |  |  |  |  |
| Tipu | 17 | –9.9 | 0.8 | +9.2 | 0.5 | 4 | –9.5 | 2.5 | +9.4 | 1.2 |
| Xunantunich/  San Lorenzo | 8 | –11.0 | 1.6 | +9.5 | 0.9 |  |  |  |  |  |
| **Total** | **65** | –**10.3** | **1.6** | **+8.9** | **0.8** | **12** | –**10.1** | **2.1** | **+9.7** | **1.3** |

**Table S3-5. Mean** ***δ*^34^S values for human bone collagen by age category for sites where these data are available.**

| **Site** | **Adult** | | | **Sub-adult** | | |
| --- | --- | --- | --- | --- | --- | --- |
|  | ***n*** | ***δ*^34^S**  **(‰ VCDT)** | **SD** | ***n*** | ***δ*^34^S**  **(‰ VCDT)** | **SD** |
| Baking Pot | 1 | +15.9 | -- | 1 | +12.3 | -- |
| Barton Creek |  |  |  | 1 | +12.2 | -- |
| Cahal Pech | 9 | +13.8 | 0.7 | 4 | +14.0 | 1.8 |
| Caledonia | 17 | +11.1 | 2.0 | 1 | +9.4 | -- |
| Lower Dover | 1 | +10.0 | -- |  |  |  |
| Pacbitun | 8 | +12.7 | 1.0 | 1 | +12.5 | -- |
| Pook's Hill | 4 | +10.0 |  |  |  |  |
| Tipu | 17 | +12.9 | 1.1 | 4 | +11.9 | 1.5 |
| Xunantunich/ San Lorenzo | 8 | +13.7 | 1.4 |  |  |  |
| **Total** | **65** | **+12.4** | **1.8** | **12** | **+12.5** | **1.8** |

**Table S3-6. Results of statistical analyses for isotopic values by adults vs. subadults.** Numbers highlighted in green indicate statistically significant values.

| **Group** | ***n*** | **Variables** | ***t-*test (*p*)** | **Mann-Whitney U (*p*)** |
| --- | --- | --- | --- | --- |
| Adults vs. Subadults (incl. infants) | 65;12 | *δ*^13^C | 0.378 | 0.333 |
|  |  | *δ*^15^N | **< 0.05** | **< 0.05** |
|  |  | *δ*^34^S | 0.446 | 0.421 |

**Table S3-7.** **Mean *δ*^13^C and *δ*^15^N values for human bone collagen by biological sex for sites where these data are available.**

| **Site** | **Female** | | | | | **Male** | | | | |
| --- | --- | --- | --- | --- | --- | --- | --- | --- | --- | --- |
|  | ***n*** | ***δ*^13^C**  **(‰ VPDB)** | **SD** | ***δ*^15^N**  **(‰ Atm N_2_)** | **SD** | ***n*** | ***δ*^13^C**  **(‰ VPDB)** | **SD** | ***δ*^15^N**  **(‰ Atm N_2_)** | **SD** |
| Cahal Pech | 2 | –12.2 | 0.6 | +8.1 | 0.5 | 3 | –11.2 | 1.8 | +9.3 | 0.9 |
| Caledonia | 1 | –12.3 | -- | +8.6 | -- | 1 | –11.1 | -- | +8.8 | -- |
| Lower Dover |  |  |  |  |  | 1 | –9.5 | -- | +8.7 | -- |
| Pacbitun | 5 | –10.2 | 2.2 | +8.8 | 0.4 | 3 | –9.4 | 0.6 | +9.2 | 1.2 |
| Pook's Hill |  |  |  |  |  | 2 | –10.7 | 0.0 | +8.6 | 0.2 |
| Tipu | 6 | –9.6 | 0.5 | +9.0 | 0.5 | 8 | –9.8 | 1.0 | +9.4 | 0.5 |
| Xunantunich/  San Lorenzo | 2 | –10.8 | 2.1 | +9.8 | 0.4 | 4 | –11.7 | 1.7 | +9.4 | 1.1 |
| **Total** | **16** | –**10.4** | **1.6** | **+8.9** | **0.6** | **22** | –**10.4** | **1.4** | **+9.2** | **0.7** |

**Table S3-8.** **Mean *δ*^34^S values for human bone collagen by biological sex for sites where these data are available.**

| **Site** | **Female** | | | **Male** | | |
| --- | --- | --- | --- | --- | --- | --- |
|  | ***n*** | ***δ*^34^S**  **(‰ VCDT)** | **SD** | ***n*** | ***δ*^34^S**  **(‰ VCDT)** | **SD** |
| Cahal Pech | 2 | +13.3 | 0.7 | 3 | +14.1 | 0.5 |
| Caledonia | 1 | +11.8 | -- | 1 | +9.9 | -- |
| Lower Dover |  |  |  | 1 | +10.0 | -- |
| Pacbitun | 6 | +12.9 | 1.2 | 3 | +12.4 | 0.6 |
| Pook's Hill |  |  |  | 2 | +10.4 | 0.8 |
| Tipu | 6 | +13.1 | 1.1 | 8 | +13.1 | 0.9 |
| Xunantunich/ San Lorenzo | 2 | +12.3 | 1.6 | 4 | +14.4 | 1.3 |
| **Total** | **16** | **+12.9** | **1.1** | **22** | **+12.8** | **1.7** |

**Table S3-9. Results of statistical analyses for isotopic values by females vs. males.**

| **Group** | ***n*** | **Variables** | ***t-*test (*p*)** | **Mann-Whitney U (*p*)** |
| --- | --- | --- | --- | --- |
| Females vs. Males | 16;22 | *δ*^13^C | 0.481 | 0.500 |
|  |  | *δ*^15^N | 0.055 | 0.107 |
|  |  | *δ*^34^S | 0.445 | 0.429 |

**Sulfur Group Analyses**

To investigate the relationship between diet and geographic location, samples were assigned sulfur groups. Plots comparing mean *δ*^34^S values from each site were created to illustrate statistical relationships between sites, with each site treated independently (Fig 5 in text). Error ranges for 80%, 95%, and 99% confidence intervals (CIs) were calculated separately on the basis of each sample for sites with >5 individuals whose sulfur measurements met quality control standards (after Drennan 2009:149-151). The sample from Caracol did not include the extreme outlier identified. Sulfur groups included sites with mean values that varied significantly at the 99% CI.

**Table S3-10.** **Comparison of *δ*^34^S values in ‰ by site.** Sites listed corresponding to Figure 5 in text, with means in descending order.

| **Site** | **Sample Size** | **Mean** | **80% CI** | **90% CI** | **95% CI** |
| --- | --- | --- | --- | --- | --- |
| Cahal Pech | 15 | +13.8 | +13.8 ± 0.4 | +13.8 ± 0.6 | +13.8 ± 0.8 |
| Xunantunich/San Lorenzo | 8 | +13.7 | +13.7 ± 0.7 | +13.7 ± 1.1 | +13.7 ± 1.7 |
| Baking Pot | 3 | +12.6 | -- | -- | -- |
| Tipu | 21 | +12.7 | +12.7 ± 0.3 | +12.7 ± 0.5 | +12.7 ± 0.7 |
| Pacbitun | 10 | +12.6 | +12.6 ± 0.4 | +12.6 ± 0.7 | +12.6 ± 0.9 |
| Caledonia | 22 | +11.2 | +11.2 ± 0.5 | +11.2 ± 0.8 | +11.2 ± 1.1 |
| Caracol | 15 | +11.1 | +11.1 ± 0.7 | +11.1 ± 1.0 | +11.1 ± 1.4 |
| Lower Dover | 2 | +10.5 | -- | -- | -- |
| Pook's | 6 | +10.0 | +10.0 ± 0.4 | +10.0 ± 0.7 | +10.0 ± 1.1 |
| Maya Mts | 2 | +8.5 | -- | -- | -- |

At least two groups could confidently be identified. Group 1 consisted of Belize Valley and Belize Valley southern periphery sites (Table S3-11 and Figure S3-3). Group 2 included sites primary from the Vaca Plateau, with one site from the Belize Valley and one sites from the southern periphery. Group 1 has a significantly higher mean *δ*^34^S values compared to Group 2 (*t* = 6.58; *df* = 77; *p* > 0.001), general corresponding with geology. We estimate that mean *δ*^34^S values for individuals from Group 1 sites is 13.1 ± 0.5 ‰ at the 99% CI. Our estimate for Group 2 *δ*^34^S values is 10.7 ± 1.0 ‰ at the 99% CI. A possible third group may also exist, but because of the small sample size (n=2; Maya Mountain sites), statistical analyses could not be performed to clearly identify it as a distinct group. There were no strong or statistically significant correlations between *δ*^15^N and *δ*^34^S values for any sulfur group (Tables S21).

**Table S3-11. Comparison of *δ*^34^S values by sulfur group.**

| **Confidence Level** | **Mean *δ*^34^S (‰)** | |
| --- | --- | --- |
|  | **Group 1** | **Group 2** |
| 80% | +13.1 ± 0.2 | 10.7 ± 0.5 |
| 95% | +13.1 ± 0.3 | 10.7 ± 0.8 |
| 99% | +13.1 ± 0.5 | 10.7 ± 1.0 |


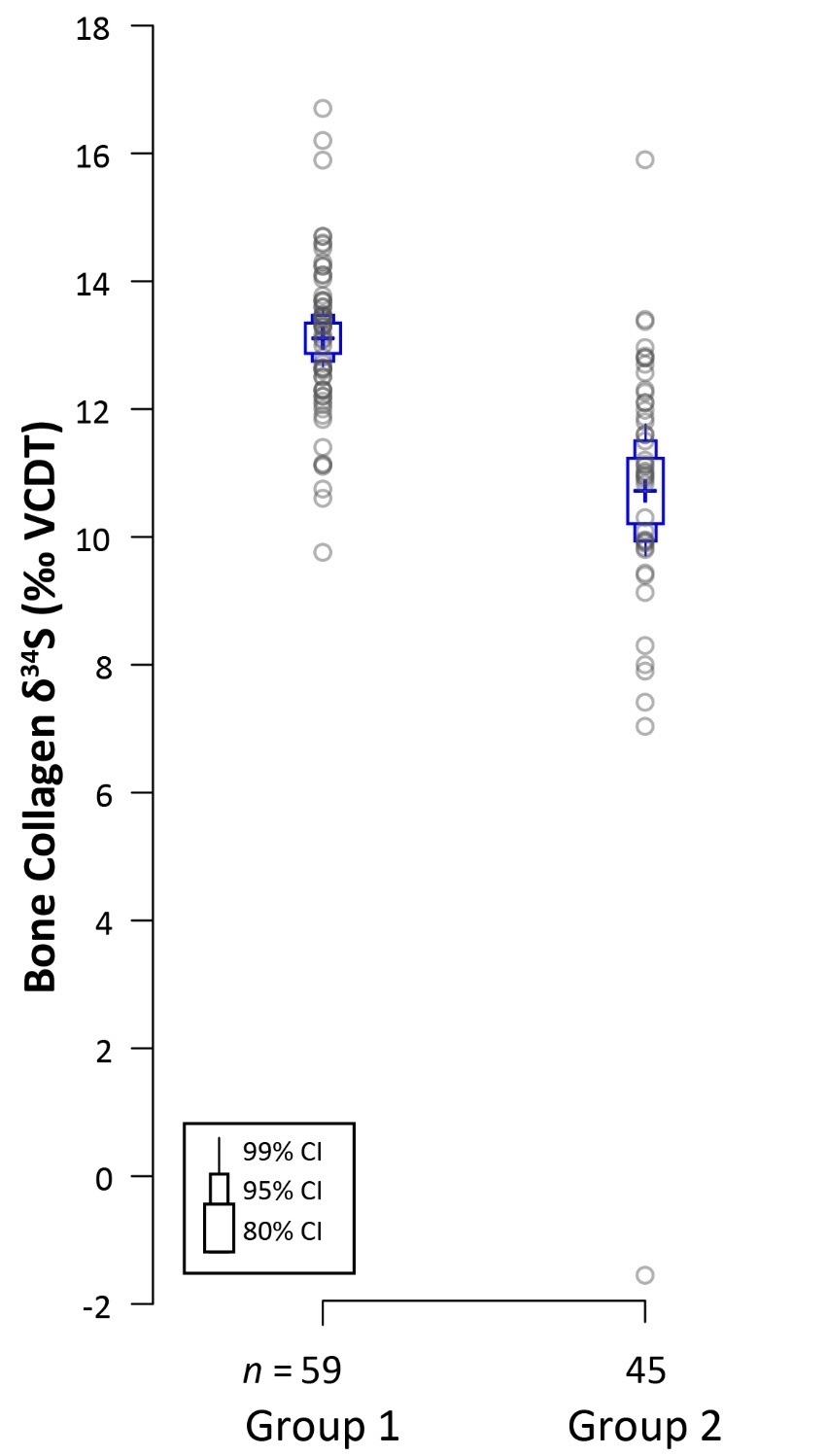


**Figure S3-3. Bullet graph of stable sulfur (*δ*^34^S) isotope values for human bone collagen plotted by sulfur groups.** Data points are shown as circles with the means for each time period indicated by a blue cross. The bullet graph shows the 80%, 95%, and 99% confidence intervals (CIs; thickest to thinnest bullets) around the mean corresponding with Table S3-11.

**Multivariate Statistical Analyses**

Multivariate analyses for isotopic data (*δ*^13^C, *δ*^15^N, and *δ*^34^S) are described below. Pearson’s *r* was used to measure of the strength of linear relationships (both positive and negative) between paired datasets (*δ*^13^C and *δ*^34^S; *δ*^15^N and *δ*^34^S). The non-parametric Spearman’s rank correlation (*r_s_*) was also used to determine correlations between *δ*^13^C, *δ*^15^N, and *δ*^34^S values. Data was ranked by 1) high *δ*^13^C values and *δ*^34^S values, indicating a correlation between C_4_ food consumption and sulfur, and 2) high *δ*^15^N values and *δ*^34^S values, indicating correlation between meat/marine protein consumption and sulfur. Spearman’s rank correlation was chosen because the data are not normally distributed. Additionally, this test does not require transformations or other modifications (e.g., removal of outliers) within the dataset (Drennan 2009:228), which can skew Pearson’s r results. Rank order relationships (i.e., correlation strength) were assigned values between 1 and −1, indicating positive and negative correlation, respectively (Drennan 2009:224).

When considering the entire dataset, there was no statistically significant relationship between *δ*^13^C and δ^34^S values (r = 0.0023, *p* = 0.981) or *δ*^15^N and *δ*^34^S values (*r* = -0.0667, *p* = 0.510). Additionally, there is only a weak statistically insignificant rank-order correlation between *δ*^13^C and *δ*^34^S values (*r_s_* = 0.025, *p* = 0.802) and between δ^15^N and δ^34^S values (*r_s_* = -0.038, *p* = 0.704). Multivariate analyses also include comparisons at the site-level and within defined sulfur groups, which are described below.

**Site-level analyses**

When the data were examined at the site-level, there are relatively weak correlations between *δ*^13^C and *δ*^34^S values (Table S3-12) and between *δ*^15^N and *δ*^34^S values (Table S3-13). The exception is the site of Caledonia, where there is a statistically significant strong positive correlation between *δ*^13^C and *δ*^34^S values (*r* = 0.703, *p* < 0.001; *r_s_* = 0.681, *p* < 0.05). A significant positive correlation between *δ*^15^N and *δ*^34^S values (*r* = 0.544, *p* < 0.05), though the rank-order correlation is moderate (not statistically significant).

**Table S3-12. Results of Pearson’s *r* and Spearman’s rank correlation (*r_s_*) for *δ*^13^C and *δ*^34^S values at the site level.** Numbers highlighted in green indicate strong correlation and statistically significant values.

| **Site** | ***n*** | **Pearson’s *r*** | ***p*** | **Spearman’s rank**  **correlation (*r_s_*)** | ***p*** | **Rank Correlation** |
| --- | --- | --- | --- | --- | --- | --- |
| Baking Pot | 3 | -- | -- | -- | -- | -- |
| Barton Creek | 1 | -- | -- | -- | -- | -- |
| Cahal Pech | 15 | 0.064 | 0.821 | -0.002 | 0.994 | very weak |
| Caledonia | 18 | **0.703** | **< 0.01** | **0.681** | **< 0.05** | **strong** |
| Caracol | 15 | -0.345 | 0.208 | -0.209 | 0.474 | weak |
| Lower Dover | 2 | -- | -- | -- | -- | -- |
| Maya Mountains Sites | 2 | -- | -- | -- | -- | -- |
| Pacbitun | 10 | -0.544 | 0.104 | -0.329 | 0.352 | weak |
| Pook's Hill | 6 | 0.737 | 0.095 | 0.800 | 0.104 | very strong |
| Tipu | 21 | -0.0365 | 0.877 | -0.024 | 0.920 | very weak |
| Xunantunich/  San Lorenzo | 8 | 0.002 | 0.996 | -0.036 | 0.939 | very weak |

**Table S3-13. Results of Pearson’s *r* and Spearman’s rank correlation (*r_s_*) for *δ*^15^N and *δ*^34^S at the site level.** Numbers highlighted in green indicate strong correlation and statistically significant values.

| **Site** | ***n*** | **Pearson’s *r*** | ***p*** | **Spearman’s rank**  **correlation (*r_s_*)** | ***p*** | **Rank**  **Correlation** |
| --- | --- | --- | --- | --- | --- | --- |
| Baking Pot | 3 | -- | -- | -- | -- | -- |
| Barton Creek | 1 | -- | -- | -- | -- | -- |
| Cahal Pech | 15 | 0.043 | 0.879 | 0.213 | 0.464 | weak |
| Caledonia | 18 | **0.544** | **< 0.05** | 0.463 | 0.061 | moderate |
| Caracol | 15 | -0.247 | 0.375 | -0.279 | 0.334 | weak |
| Lower Dover | 2 | -- | -- | -- | -- | -- |
| Maya Mountains Sites | 2 | -- | -- | -- | -- | -- |
| Pacbitun | 10 | 0.003 | 0.993 | -0.018 | 0.960 | very weak |
| Pook's Hill | 6 | 0.388 | 0.447 | 0.300 | 0.623 | weak |
| Tipu | 21 | -0.301 | 0.184 | -0.334 | 0.150 | weak |
| Xunantunich/  San Lorenzo | 8 | -0.097 | 0.819 | -0.036 | 0.938 | very weak |

**Time period**

The results of multivariate statistical analyses by time period are presented in Tables S3-14 and S3-15. There were no statistically significant correlations detected between *δ*^13^C and *δ*^34^S values or *δ*^15^N and *δ*^34^S values for any time period. Correlations between *δ*^13^C and *δ*^34^S values are relatively weak through time, though there is a moderate negative correlation (not statistically significant) during the Late Preclassic, perhaps reflecting changes in diet for some individuals (i.e., increased maize consumption) for the period, though it should be noted that four of five Late Preclassic samples are from Cahal Pech, which has a higher *δ*^34^S signature compared to other sites. Additionally, there is a positive statistically significant correlation between *δ*^13^C and *δ*^34^S values for the Late Classic, though the correlation is weak. Moderate negative correlations (not statistically significant) are also present between *δ*^15^N and *δ*^34^S values during the Early Classic. These correlations indicate that higher *δ*^15^N values are correlated with lower *δ*^34^S values.

**Table S3-14. Results of Pearson’s *r* and Spearman’s rank correlation (*r_s_*) for *δ*^13^C and *δ*^34^S values by time period.**

| **Time Period** | ***n*** | **Pearson’s *r*** | ***p*** | **Spearman’s rank**  **correlation (*r_s_*)** | ***p*** | **Rank Correlation** |
| --- | --- | --- | --- | --- | --- | --- |
| Late Preclassic | 5 | -0.696 | 0.395 | -0.600 | 0.284 | moderate |
| Early Classic | 7 | 0.370 | 0.413 | 0.357 | 0.432 | weak |
| Late Classic | 53 | 0.144 | 0.301 | **0.285** | **0.038** | **weak** |
| Terminal Classic | 13 | -0.188 | 0.538 | -0.330 | 0.271 | weak |
| Postclassic | 11 | -0.274 | 0.415 | -0.104 | 0.759 | weak |
| Colonial | 11 | 0.008 | 0.981 | -0.100 | 0.770 | weak |

**Table S3-15. Results of Pearson’s *r* and Spearman’s rank correlation (*r_s_*) for *δ*^15^N and *δ*^34^S values by time period.**

| **Time Period** | ***n*** | **Pearson’s *r*** | ***p*** | **Spearman’s rank**  **correlation (*r_s_*)** | ***p*** | **Rank Correlation** |
| --- | --- | --- | --- | --- | --- | --- |
| Late Preclassic | 5 | -0.193 | 0.757 | -0.200 | 0.747 | weak |
| Early Classic | 7 | -0.544 | 0.207 | -0.558 | 0.192 | moderate |
| Late Classic | 53 | 0.069 | 0.620 | 0.145 | 0.299 | weak |
| Terminal Classic | 13 | -0.245 | 0.419 | -0.335 | 0.263 | weak |
| Postclassic | 11 | -0.348 | 0.294 | -0.145 | 0.667 | weak |
| Colonial | 11 | -0.351 | 0.289 | -0.190 | 0.572 | weak |

**Age and Sex**

We also examined if there was an age or sex effect influencing correlations between isotope values. As noted above, the sample for which age could be determined including 12 sub-adults (including 3 infants) and 66 adults. The results of Pearson’s *r* and Spearman’s rank correlation analyses is presented in Tables S3-16 and S3-17. While correlations between *δ*^13^C and *δ*^34^S values were very weak for both adults and sub-adults, there is a moderate rank correlation between *δ*^15^N and *δ*^34^S values for subadults, though it is not statistically significant.

The sample analyzed by sex includes 16 females and 22 males (Tables S3-18 and S3-19). No strong or statistically significant correlations were found between *δ*^13^C and *δ*^34^S values or between *δ*^15^N and *δ*^34^S values for either females or males.

**Table S3-16. Results of Pearson’s *r* and Spearman’s rank correlation (*r_s_*) for *δ*^13^C and *δ*^34^S values by age category.**

| **Age** | ***n*** | **Pearson’s *r*** | ***p*** | **Spearman’s rank correlation (*r_s_*)** | ***p*** | **Rank Correlation** |
| --- | --- | --- | --- | --- | --- | --- |
| Adult | 65 | 0.120 | 0.341 | 0.047 | 0.710 | very weak |
| Subadult  (includes infants) | 12 | -0.014 | 0.968 | -0.119 | 0.713 | very weak |

**Table S3-17. Results of Pearson’s *r* and Spearman’s rank correlation (*r_s_*) for** ***δ*^15^N and *δ*^34^S values by age category.**

| **Age** | ***n*** | **Pearson’s *r*** | ***p*** | **Spearman’s rank correlation (*r_s_*)** | ***p*** | **Rank Correlation** |
| --- | --- | --- | --- | --- | --- | --- |
| Adult | 65 | 0.234 | 0.061 | 0.154 | 0.221 | very weak |
| Subadult  (includes infants) | 12 | -0.519 | 0.085 | -0.497 | 0.099 | moderate |

**Table S3-18. Results of Pearson’s *r* and Spearman’s rank correlation (*r_s_*) for *δ*^13^C and *δ*^34^S values by biological sex.**

| **Sex** | ***n*** | **Pearson’s *r*** | ***p*** | **Spearman’s rank correlation (*r_s_*)** | ***p*** | **Rank Correlation** |
| --- | --- | --- | --- | --- | --- | --- |
| Female | 16 | -0.391 | 0.134 | -0.345 | 0.190 | weak |
| Male | 22 | -0.051 | 0.821 | -0.052 | 0.816 | very weak |

**Table S3-19. Results of Pearson’s *r* and Spearman’s rank correlation (*r_s_*) for *δ*^15^N and *δ*^34^S values by sex.**

| **Sex** | ***n*** | **Pearson’s *r*** | ***p*** | **Spearman’s rank correlation (*r_s_*)** | ***p*** | **Rank**  **Correlation** |
| --- | --- | --- | --- | --- | --- | --- |
| Female | 16 | -0.267 | 0.317 | -0.219 | 0.415 | weak |
| Male | 22 | 0.331 | 0.132 | 0.173 | 0.439 | very weak |

**References**

Drennan RD. Statistics for Archaeologists: A Commonsense Approach, second edition. New York: Springer; 2009.

Gauthier TD, Hawley ME. Chapter 5 - Statistical Methods. In Murphy BL, Morrison RD, editors. New York: Academic Press; 2015. pp. 99-148.

Katzenberg MA, Saunders SR, Fitzgerald WR. Age differences in stable carbon and nitrogen isotope ratios in a population of prehistoric maize horticulturists. Am. J. Phys. Anthropol. 1993; 90(3): 267-281.

Reynard, LM, Tuross N. The known, the unknown and the unknowable: weaning times from archaeological bones using nitrogen isotope ratios. J. Archaeol. Sci. 2015; 53: 618-625. doi: 10.1016/j.jas.2014.11.018.

Ruxton GD. The unequal variance t-test is an underused alternative to Student's t-test and the Mann–Whitney U test. Behav. Ecol. 2006; 17 (4):688-690. doi: 10.1093/beheco/ark016

Williams JS, White CD, Longstaffe FJ. Trophic level and macronutriten shift effects associated with the weaning process in the Postclassic Maya. Am. J. Phys. Anthropol. 2005; 128:b 781-790.

Wright LE. Examining Childhood Diets at Kaminaljuyu, Guatemala, through Stable Isotopic Analysis of Sequential Enamel Microsamples. Archaeometry 2013; 55:113-133. doi: 10.1111/j.1475-4754.2012.00668.x

Wright LE, Schwarcz HP. Stable carbon and oxygen isotopes in human tooth enamel: identifying breastfeeding and weaning in prehistory. Am. J. Phys. Anthropol. 1998; 106: 1-18.
