# Supplementary material for: Sulfur isotopes as a proxy for human diet and mobility from the preclassic through colonial periods in the Eastern Maya lowlands
Source: PLoS One. 2021 Aug 12;16(8):e0254992. doi: 10.1371/journal.pone.0254992 (PMC8360522; doi:10.1371/journal.pone.0254992)
Supplement: Attachment — Submitted filename: Response to Reviewers - Ebert et al..docx [file pone.0254992.s007.docx]

**Response to Reviewers**

**Editor Comments**

1. I have also some concerns with the selection of the data considered as reliable. In the S1 Table, I noticed that you keep in Sulfur Group 2 samples with N:S lower than the 100 recommended by Nehlich and Richards (2009). As a matter of fact, these two collagen samples display a S content of 0.40 or higher. Nehlich and Richards (2009) recommend a S content range of 0.15 to 0.35% for mammal bones from archaeological contexts in addition to given C:S and N:S ranges. In Bocherens et al. 2011 (Palaeo, Paleaeo, Palaeo) the highest S content in modern mammals is found for reindeer with values as high as 0.33%. I would thus definitely not consider as secured the 34S abundances of collagen with a S content higher than 0.35%, as the authors state themselves in the supplementary data, which is the case for a number of samples of Sulfur group 2. Unless I am mistaken, it seems the authors have nevertheless not discarded these data from the final interpretation. This point needs to be addressed since all criteria of preservation have to be scrutinized and fulfilled before further elaborating on the meaning of the isotopic ratios.

To address this issue, we have eliminated additional samples from our statistical analyses, which form the basis of our discussion in the results section. We eliminated all samples that had a wt % of sulfur exceeding the range from 0.15 – 0.35 % for mammal bones recommended by Nehlich and Richards (2009). The samples included BKP09, BKP11; BKP29, BKP35. Additionally, we revaluated C:S and N:S ratios, eliminating two samples with C:S ratios outside the 600±300 (BKP15 and TP005) and three samples with N:S ratios outside 200±100 range (BKP47, BKP41, and TP492) recommended by Nehlich and Richards (2009). The remaining 102 samples met all other quality control standards, and we have updated all our statistical analyses based solely on these samples. Our new statistical data did not change our interpretations except for division of the samples in to sulfur groups (see Reviewer 1, Comment 25).

2. I am puzzled by the collagen C content of 50% or higher which is over the expected value of even like-fresh collagen. I suspect you may have had some issues with the calibration of the elemental analysis. I would encourage you to check your measures comparing with the in-house standards with known elemental composition that were run in the same analytical sets.

We have double checked the calibration standards for samples with C% greater than 50%. We note that the samples in question were not all measured for C and N at the same time, but all samples were prepared in the Penn State Human Ecology and Isotope Geochemistry lab and measured at Yale Analytical and Stable Isotope Center (YASIC). Quality assurance and normalization data from YASIC standards for these runs fall within the acceptable range accounting for measurement error. The C:N ratio for the samples falls within the acceptable ranges. Therefore, we attribute the higher-than-expected C% data to the sample being larger than the ostensible submitted mass. In other words, we believe the issue stems from the initial weighing of samples prior to analysis, likely due to static or vibrations impacting the balance. Because the proportion of C and N remains the same as percent of the sample, the wrong weight is cancelled out when considering the percentages of each, which leads to a C:N that falls within acceptable parameters.

This does impact the C:S and N:S ratios, however. As a test, we recalculated these ratios assuming expected C% (44%) and N% (16%) values. The result was that all C:S and N:S fell within the range recommended by Nehlich and Richards (2009). It is important to note we have *not* updated our reporting of these values in the paper based on these calculations. Instead, we have included a statement in the Supporting Information document noting the problem with the balance (S2 Text). This also means that we still have eliminated two samples – BKP11 and TP005 – from our statistical analysis. BKP11 remains eliminated because of a high wt% S value. Though our test calculations show that TP005 had an initial C% value well above ~50% and a N% value above ~16%, suggesting to us that other problems may exist with the quality of this sample. We consider other samples – ZTZ08, CRC19B, and TP053 – acceptable for inclusion in analyses.

Nehlich O, Richards MP. Establishing collagen quality criteria for sulphur isotope analysis of archaeological bone collagen. Archaeol. Anthropol. Sci. 2009; 1: 59-75. doi: 10.1007/s12520-009-0003-6.

**Reviewer 1 Comments**

1. In future submissions please include line numbers so that peer reviewers can spend less time pinpointing the lines to which they’re referring. In the following comments: S = section number; P = paragraph number within that section; L = line number(s) within that paragraph.

We apologize this oversight in our initial submission, and updated our revised manuscript to include numbered lines for ease of commenting.

2. S2, P2, L7. You may wish to change this slightly as it is technically more correct to say “less negative” rather than “more positive” when referring to negative values.

We have made this update in text and have also made similar changes throughout the entire paper.

3. S2, P2, L9. This is perhaps a bit simplistic – C_3_ environments have a tremendous range of *δ*^13^C variation, which can lead to systematic variation in human and animal *δ*^13^C. For instance, major axes of *δ*^13^C variation include structural differences among C_3_ environments, such canopy density (Drucker, et al. 2008; van der Merwe and Medina 1991), or dietary foci on differ plant tissues (i.e., vegetation vs. mast/nuts/seeds), which can lead to multi ‰ offsets in consumers (Guiry, et al. 2020). Given the importance of *δ*^13^C in regional diet interpretations, and the potential significance of forest ecosystems in the area, these kinds of potential sources of *δ*^13^C variation would seem relevant to mention.

We thank the reviewer for pointing out these helpful sources. Based on this comment, we have included information from these papers in the background section on stable isotope studies in the Maya lowlands. More specifically, we updated the paragraph starting on Line 125 in the “clean” version of the manuscript to add additional explanation regarding expected *δ*^13^C values in the Maya lowlands based on the consumption of C_3_ vs C_4_ vegetation. Because we do not present faunal data is this study, we are more concerned with C_4_ consumption by humans, which composted over 70% of dietary protein by 3000 BP (well at least 1000 years prior to the earliest dated burial in our study; see Kennett et al. 2020).

Kennett, DJ, Prufer KM, Culleton BM, George RJ, Robinson M, Trask WR, Buckley GM, Moes E, Kate EJ, Harper TK, O’Donnell L, Ray EE, Hill EC, Alsgaard A, Merriman C, Meredith C, Edgar HJH, Awe JJ, Gutierrez SM. Early isotopic evidence for maize as a staple grain in the Americas. Sci. Adv. 2020; 6(23): doi: 10.1126/sciadv.aba3245. doi: 10.1126/sciadv.aba3245.

4. S2, P2, Last line. Again, a bit more nuance would be useful here. In archaeology we tend to vastly oversimplify variation in marine resource *δ*^13^C, and this can lead o missed opportunities for understanding past diets. The bone collagen *δ*^13^C of aquatic species can span 10-15‰, based on the type of marine environments, and for many economically important fish *δ*^13^C can be very low indeed (i.e., easily overlapping with C_3_ diets). The ecological literature (although often focusing on other tissues) is a good place to start to get a better sense of this (e.g., Satterfield and Finney 2002). Not that you’d need to expand much here, but some acknowledgment of these potential issues would add interpretive transparency for readers with less background in biogeochemistry.

We have elaborated on this point brought up the reviewer (Lines 165-168 in “clean” manuscript) and base our comments on a recent study by Rand et al. (2021), which was not available at the time of our initial submission. This study found that Caribbean species, which would have been the marine species potentially included in Eastern lowland diets, had elevated *δ*^13^C values (even higher than maize). However, there is no strong archaeological evidence for high levels marine food consumption in this inland zone of the Maya lowlands (located ~80 km from the Caribbean coast), so we believe less negative *δ*^13^C values can be attributed more concretely to maize consumption.

Rand AJ, Freiwald C, Grimes V. A multi-isotopic (*δ*^13^C, *δ*^15^N, and *δ*^34^S) faunal baseline for Maya subsistence and migration studies. J. Archaeol. Sci. Rep. 2021; 37: doi: 10.1016/j.jasrep.2021.102977.

5. S2, P2, Last line. Wasn’t Chisholm, et al. (1982) first to look at this in an archaeological context?

We thank the reviewer for pointing us to this citation in relation to the discussion cited above in Comment 4.

6. S2, P3, L2. The 6‰ trophic enrichment factor (TEF) estimate is considered by many to be extreme. It’s a hypothetical estimate based on stacked uncertainties from combining inter-tissue offsets. Are you weighing this TEF on equal footing with the much more widely acknowledged and studied 3-4‰ estimates?

This is a comment that was also echoed by the Editor and Reviewer 2. We have revised or estimate of trophic enrichment to a range of 3-5 ‰ for terrestrial ecosystems, citing a study by Bocherens and Drucker (2003).

Bocherens H, Druker D. Trophic Level Isotopic Enrichment of Carbon and Nitrogen in Bone Collagen: Case Studies from Recent and Ancient Terrestrial Ecosystems. Int. J. Osteoarchaeol. 2003; 13: 46-53. doi: 10.1002/oa.662.

7. S2, P3, L4-6. Again some more nuance here might be helpful. Like *δ*^13^C, *δ*^15^N baselines in marine environments are highly context dependent (but at least for *δ*^15^N, there’s a really good review out there: (Sigman, et al. 2009). You’re explanation for why marine foods lead to high *δ*^15^N in consumers is not quite correct. The reason that *δ*^15^N is often assumed to be higher in marine foods than terrestrials foods (by archaeologists anyhow) has more to do with trophic level than it does baselines. Marine ecosystems can have up to 5 trophic levels while terrestrial systems top out at 3. This leads to much higher *δ*^15^N for people eating of marine animal protein from TLs 3-5.

We have expanded this section, making it a stand-alone paragraph (Lines 145-155). In addition to updating the description for why marine foods are expected to have higher *δ*^15^N values, we also describe published nitrogen data from the Maya lowlands that demonstrate that *δ*^15^N values of marine fauna overlap with those of both wild and terrestrial animals, resulting in human bone collagen values that mimic those of maize-based diet.

8. Consumption of marine foods at TLs 1 or 2 won’t necessarily lead to higher bone collagen *δ*^15^N.

This point is well taken, and we have clarified that higher bone collagen *δ*^15^N is expected for humans who consume marine resources specifically from higher trophic levels.

9. S2, P3, L7-8. Archaeologists widely assume that freshwater fish *δ*^13^C should be lower, and this is certainly true for select species in some areas (we even get bone collagen *δ*^13^C in the –30s in some regions), but it not necessary the case everywhere. Guiry 2019, which you cite here, is actually cautioning archaeologists to pay more attention to this variation, especially potential sources of higher *δ*^13^C in freshwater foods – which can happen when fish are sourced from certain places like nearshore areas, higher productivity ecosystems, and potentially areas where aquatic carbon budgets may be influenced by CO_2_ subsidies from ancient marine sedimentary substrates. If you don’t have data from archaeological fish in hand, you may want to explore whether it’s likely that relevant fish taxa in the area would likely have had higher or lower *δ*^13^C based on local geography/ecology of applicable watersheds. Also, rather than being tucked into a line about *δ*^15^N, potential issues about variation in freshwater resources *δ*^13^C should be dealt with in the preceding paragraph, and given some dedicated attention (again, especially given the importance of *δ*^13^C for interpreting overall trends in diet in the region).

We have expanded our discussion of isotopic values for freshwater foods, focusing on both *δ*^13^C and *δ*^15^N data specific to the Maya lowlands. We have also cited the few available studies (only two) that have published *δ*^13^C and *δ*^15^N isotope values in freshwater fish in the Maya lowlands (Rand et al. 2021; Wright 2006) to better characterize the expected variability in our human bone collagen data.

Rand AJ, Freiwald C, Grimes V. A multi-isotopic (*δ*^13^C, *δ*^15^N, and *δ*^34^S) faunal baseline for Maya subsistence and migration studies. J. Archaeol. Sci. Rep. 2021; 37: doi: 10.1016/j.jasrep.2021.102977.

Wright LE. Diet, health and status among the Passion Maya: a reappraisal of the collapse. Nashville: Vanderbilt University Press; 2006.

10. S2, P3, Last line. The potential importance of agricultural impacts on *δ*^15^N should also be acknowledged, especially in this region. For an excellent review on this, see Szpak (2014). It may also be worth considering the potential impacts of agriculture on *δ*^34^S as well (Szpak, et al. 2019).

In response to this suggestion, we have also expanded our discussion of the cultural factors that could impact *δ*^15^N values, specifically slash-and-burn (i.e. *milpa* farming, starting on Line 169). We suggest that these factors would most likely influence the *δ*^15^N values of humans who relied heavily on domesticates from *milpa* farming, as there is little convincing isotopic evidence for animals (e.g. deer) consuming domesticated plants in large quantities (or at least enough to be detected isotopically). We also thank the reviewer for pointing us to the Szpak references. We comment on the impact of agriculture on sulfur isotope values beginning on Line 175.

11. S2. P4, L10-13. This has now been empirically demonstrated within a modern experimental context using bone collagen (Guiry and Szpak 2020).

We thank the reviewer for providing this citation and have included it in the discussion referenced.

12. S2. P4, L13-15. I’m not sure to what extent estuarine resources could be relevant here, but it might also be worth noting that some ‘freshwater’ resources from areas that receive minimal seawater inputs can still appear ‘marine’ in their *δ*^34^S. This is because allochthonous seawater S inputs, due to the much higher S content of seawater relative to freshwater, overwhelms autochthonous freshwater S sources (Fry and Chumchal 2011). This can mean that species you’d assume were freshwater, will contribute a ‘marine’ *δ*^34^S signal to diet.

Though costal/estuarine resources were likely not important components of the diets in the study region (located ~80 km from the nearest coastline), we mention this potentially confounding factor as it might be important for those working in other regions of the Maya lowlands closer to the coast.

13. Something you begin to allude to here, but which can (and I’d argue really should) be considered in more detail is the potential importance of sulfide-derived S in wetland resources. A recent paper has demonstrated a very large impact of consuming sulfide tolerant plants (or plants in areas where there is more sulfate from reoxidised sulfide) on consumer bone collagen *δ*^34^S (Guiry, et al. 2021). While this archaeological study was focusing on costal marshes, the principles at play may also apply at inland wetlands as well (early data from (Cornwell, et al. 1995) suggests this). As outlined in the 2021 paper, the underlying mechanisms that leads to lower *δ*^34^S in wetlands (anoxia) may also result in higher *δ*^15^N (due to coupled nitrification-denitrification in marsh adapted rhizomes) for affected plants and their consumers. This means that you could be able to assess the potential for this kind of resource consumption in your samples with the data you already have. My feeling though is that the broad scale diet of humans might serve to obscure patterns, but this may, at least, help you assess what is going on with your low *δ*^34^S outlier (see Comment 21).

Based on the recent Guiry et al. (2021) paper mentioned by the reviewer, we have expanded our discussion of the potential for wetland resource consumption. A confounding factor in this study, and indeed all studies of archaeological *δ*^34^S in the Maya lowlands is the absence of baseline data. A recent publication by Rand et al. (2021; published April 2021) provides the only systematically collected faunal baselines data for the entire Maya region. These data suggest a range of values from approximately +2 to +16 ‰, reflecting a combination of sample location (coastal vs. inland) and the feeding habits of different species (turtle, crocodiles, limpkins). Without baselines data from additional species from the study region, it remains unclear how lacustrine or freshwater foods are reflected in the *δ*^34^S values of human bone collagen.

14. S3.1, P1, Last line. This seem like a slight over generalization. As per Comment 12, the importance of wetland resource could have a meaningful impact and you could do more to unpack this or at least acknowledge that fact that large uncertainties remain in your ability to anticipate/untangle the relative importance of geological vs. dietary (between the poles of marine- and sulphide-influenced *δ*^34^S) source of sulfur isotope variation. As it stands, based on this statement, I think readers without a strong background could misunderstand the degree of confidence we can have in these kinds of data.

We agree that environmental sulfur isotope values are complex and are influenced by multiple inputs, including geological erosion, atmospheric deposits, and sulfate-reducing bacteria in anoxic conditions; however, the contributions of these sources to environmental sulfur isotope values or those of Maya bone collagen require additional baseline studies that are currently unavailable for our study area. To address this statement, we have added more detail about the potential sources of sulfur in our study area and explained why we consider geological inputs to be most significant, including the recent faunal baseline study by Rand et al. (2021).

15. S3.2. You go into extensive site-by-site detail here, which is good. Reading the paper, I felt that this was a bit out of balance with the detail/attention you give to isotope ecology. I point this out here to support the above and below suggestions for giving closer consideration to sources of uncertainty in isotopic interpretations in general. These may be critical in terms of interpretive transparency for many of your readers who are not isotope specialists, to help them really grasp the limits of what these data can mean.

Based on this comment, and several comments above, we have greatly expanded the section on stable isotopes and paleodietary reconstruction in the background portion of the paper.

16. S4.2, P1, L3. I’m not sure you’re citing Szpak correctly here. The paper that appears in the ‘literature cited’ section is about reporting and calibration but you appear to be referring to procedures for collagen extraction here. Do you to cite Szpak’s NaOH study? (Szpak, et al. 2017)

The reviewer is correct. We included the corrected Spzak et al. 2017 citation and have updated the reference to the correct citation information. We additionally cite a second study 2017 by Spzak et al. that describes calibration and reporting of stable isotopic data, and have made clear distinctions between the two in text (see references below for Spzak et al. 2017 and Spzak et al. 2017b, respectively).

Szpak P, Krippner K, Richards MP. Effects of Sodium Hydroxide Treatment and Ultrafiltration on the Removal of Humic Contaminants from Archaeological Bone. Int. J. Osteoarchaeol. 2017; 27:1070-1077. doi: 10.1002/oa.2630.

Szpak P, Metcalfe JZ, Macdonald RA. Best practices for calibrating and reporting stable isotope measurements in archaeology. J. Archaeol. Sci. Rep. 2017; 13:609-616. doi: 10.1016/j.jasrep.2017.05.007.

17. S4.2, P3, L2. I’d encourage you to report C:N to the second decimal place here and elsewhere. Note that while precision/accuracy of the %C and % N measurements that are used to calculate C:N ratios are typically not accurate to the second, or even first, decimal place, the ratio between %C and %N definitely should be. Moreover, the second decimal place matters here – meaningful isotopic differences can occur due to contamination that is detectable even at this minute scale of variation in C:N.

All C:N ratios, where reported (e.g. Table S1) and discussed in text, have been updated to the second decimal place. We appreciate Reviewer 1 pointing out this important detail.

18. S4.2, P3. I do not have a problem with this, but technically the QC criteria you’re reporting here are part of the results and journals often require this kind of information to be placed in the results section rather than methods. One could say the same for the standard uncertainty calculation in the preceding paragraph (but for some reason, and in contrast to my comment here on collagen QC, these are typically reported in the analytical methods section).

We appreciate the reviewer’s suggestion but have decided to leave the paragraph about quality control in the Methods section as the result section primarily describes statistical analysis of the data. We are happy to rearrange this information at the discretion of the Editor.

19. Table 3. Are these “*n*=” values for the *δ*^34^S data? If so, why does Pacbitun not have a SD reported? If, not, please report these here. Also, in Section 5, Paragraph 1, you note that relevant citations for isotopic data are given in the SI. Why cite then Ebert 2019 under Table 3 as well then?

We thank the reviewer for pointing out this missing information. It appears that this table has suffered from at least two typographic errors, including that pointed out by the reviewer. This table has been updated to amend missing/erroneous data but also reflect the changes related to the samples included in analyses after quality control (see more in Editor’s Comments section above). We have also removed the citation listed under the table since it is presented in the supporting documentation, as noted by the reviewer.

20. S5.1, P2, Last few lines. Trophic level is only one of several archaeologically meaningful variables that could be at play here. There’s a lot more you could consider in this context in terms of unpacking these other sources of *δ*^15^N – not least the potential influence of agriculture on N cycling/soil-plant *δ*^15^N as per Comment 9. Readers may wonder why these issues/alternatives are not discussed.

We agree with the reviewer that there are many complex and interrelated factors at play that may influence human nitrogen isotope values. These factors have now been explored in the background section with appropriate citations, and so for brevity and to reduce repetition, we only note the potential factors that may explain the variability in human nitrogen isotope values here. We return to and expand our discussion of other potential factors influencing *δ*^15^N including agricultural (e.g. slash-and-burn *milpa* farming), vegetation (e.g. canopy effect), and climate drivers in (e.g. drought) the discussion section.

21. S5.1, P3, L3. Be careful in your terminology here with phrases like “nitrogen values”. While I do understand what you mean, this term could refer to either elemental of isotopic compositions and can therefore lead to reader confusion/uncertainty.

Based on this comment we have worked to clarify our terminology throughout the text. In particularly we have updated this section to use delta notation when referring to values of stable isotope ratios (e.g. *δ*^15^N values instead of “nitrogen values”).

22. S5.1, P4, L3. Be sure to define your acronyms (IQR), even if they should be obvious.

In this instance, and throughout the text, we have worked to ensure that all acronyms are defined at their first appearance.

23. S5.2, P1, L14. I’ve seen this in other papers and have always wondered what empirical basis it has (i.e., the idea that a sample falling a certain number of SDs away from a group is statistically meaningful). I may just be less well-versed in this approach to finding meaning in a dataset. But if I am left wondering about this question, other readers may be too and it might be worth very briefly unpacking the math or modelling that this approach is based on.

This has been updated from the original version of the paper. After re-analysis of the dataset, we decided to evaluate our results in a slightly different way. Instead of showing box plots that show standard deviations, we have illustrated the data using bullet graphs. Plots comparing mean δ^34^S values from each site were created to illustrate statistical relationships between sites, with each site treated independently (Figure 5 in text). Error ranges for 80%, 95%, and 99% confidence intervals (CIs) were calculated separately on the basis of each sample for sites with >5 individuals whose sulfur measurements met quality control standards. The graphs, along side statistical analyses presented in the Supporting Information document (S3 Text), facilitate comparisons between sample means as well as the confidence implications of their various error ranges (Drennan 2009:149-151). We find this approach more helpful than standard boxplot since bullet graph represent implications that the samples have about the populations (in this case by site) they were selected from.

Drennan RD. Statistics for Archaeologists: A Commonsense Approach, second edition. New York: Springer; 2009.

24. S5.2, P1, L4-to-end-of-paragraph. It would worth re-exploring these lower *δ*^34^S in the context of Comment 12. If the lower *δ*^34^S co-occur with higher *δ*^15^N, it could be stronger evidence that sulfide-derived S is behind these low values (indicating anoxic/wetland production). Also, I would be carful in offering bacterial sulfate reduction alone as an explanation. The product of this is sulfide, which is toxic to most plants. Low *δ*^34^S from sulfide would not automatically be reintroduced to a food web – one of two things still needs to happen: either, 1) reoxidation of sulfide-S to sulfate, which is then somehow injected back in a food web; or, 2) presence of sulfide tolerant plants (i.e., with symbiotic relationships with sulfur-oxidizing bacteria or that may themselves be chemolithotrophic). The latter is a question mark for sure, but these may be more prevalent in wet lands. In any case, the fact that bacterial sulfate reduction creates a source of isotopically light S, does not in and of itself provide a plausible source for low *δ*^34^S values.

We thank the reviewer for suggesting we examine the significance of this unusually low sulfur isotope value in more detail. While the *δ*^15^N value of this individual (+10.0 ‰) is slightly above average, it still falls within one standard deviation of the mean, and so is not elevated relative than others in the study. This suggests it is unlikely that this individual was more reliant on wetland resources than others at Caracol, or in the Eastern Maya lowlands more broadly. We discuss this in our results section. We also eliminated diagenesis as a possible explanation and retained our interpretation that this individual likely originated from beyond the Maya lowlands. Because only one sample with this extremely low value is present in our dataset, and given the fact that we do not have baseline faunal data for the site, we feel is it necessary to collect additional data before we can draw conclusions or offer other possible explanations.

25. S5.2, P3, L2. Here and elsewhere, please add “*n*=” statements in brackets for each group you compare. Please also add the mean and SD for isotope compositions in each group in brackets. This saves a lot of work for the reader.

We have made these changes throughout the text.

26. S5.2, P3, Last line. No worries either way here, but if geographical bias was a likely finding from the get-go, one could ask why make these temporal comparisons? Might they be better positioned in the SI?

Based on this comment, we have moved this lengthier discussion to the Supporting Information document and simplified our description in text.

27. S6, P4, Last two lines. Following on Comment 9, there are potentially a lot of other reasons a play here, like N cycle openness and fertilization, which can lead to multi ‰ increases in *δ*^15^N of consumer collagen through time (Guiry, et al. 2018). It would at least be worth considering the potential impacts of agriculture, in addition to land clearance.

Based on Comment 9, and our additional discussion of the potential impacts of *milpa* (slash-and-burn) agriculture on *δ*^15^N values, we have reframed this part of the discussion to consider not only forest loss and climate (i.e. environmental shifts) but also agricultural practices themselves (cultural factors) that might be at play with increasingly *δ*^15^N values in the Late/Terminal Classic in the Eastern lowlands. We decided not to include a lengthy discussion of fertilization of soils beyond *milpa* farming since this has not been directly examines by archaeologists in the study area.

28. S6, P5, L3-6. Our baselines for marine fish are fairly biased to select European species, particularly from an archaeological perspective. There are marine ecosystems (including in the Gulf of Mexico) where food webs are heavily influence by sulphide-derived S (e.g., Chasar, et al. 2005) – this is especially the case in some seagrass areas (Fry, et al. 1982; Holmer and Hasler-Sheetal 2014), due to the importance (like in wetlands) of sulfide-derived S in primary production. I’m not sure of the extent to which seagrasses are ecologically important in your broader study area, but it is worth bearing in mind that fully marine fish can have very non-‘marine’ *δ*^34^S.

As our study focused on inland sites, it is unlikely marine resources were consumed in significant quantities by the individuals included in this study. However, this may be important for future research that examines sulfur isotope values at sites closer to the Caribbean coast. Therefore, we have revised our discussion to explain that, in conjunction with carbon and nitrogen isotope data, sulfur isotope values may be more useful for distinguishing freshwater fish consumption, rather than marine resource consumption among the Maya. We have also expanded our discussion of marine *δ*^34^S values throughout the text. Although *δ*^34^S values of marine and terrestrial fauna overlap at Maya sites, preliminary data suggest terrestrial consumers may exhibit elevated *δ*^34^S values relative to those dependent on freshwater resources (Rand et al. 2021).

29. S6, P5, Last line. Can you outline why consumption of domesticates would lead to a higher *δ*^15^N? There are plenty of reason (e.g., Comments 9 and 26), but given that these animals are presumably of a similar trophic level to locally available wild animals, readers may be confused.

Multiple lines of evidence suggest that Precolonial Maya diets were heavily dependent on plant domesticates supplemented with some animal protein. In this case, we suggest that during the Colonial period, there may have been an increased consumption of animal protein generally, which would be focused on domesticated animals (e.g. cows and pigs) in lieu of wild animals obtained through hunting. This would lead to a higher *δ*^15^N values. We have clarified this in text.

30. S6, P7, L 14-20ish. The suggestion of this being evidence for a TEF for *δ*^34^S is really hypothetical and speculative. This is not an issue in and of itself, but there could be any number of reasons for the differences your seeing here, some of which (like there being a real dietary difference between infants and adults) seem more plausible to me than the idea that humans have some kind of unique fractionation for S isotopes. Bone collagen S comes from methionine, which is an essential amino acid for humans and, without offering some kind of biochemical explanation or pathway for what could plausibly explain such a TEF, it really seems like this is beyond the pale, or just too speculative to foreground like this here (let alone calling it a ‘tentative conclusion’). If you’re going to include this discussion, please add a strong caveat noting that: 1) there are/may be potentially more plausible/parsimonious explanations (e.g., diet differed); and 2) in the context of existing literature, some plausible explanation for how/why humans could have a substantial sulfur isotope TEF while other mammal species do not.

Based on re-analysis of our dataset, after eliminating additional samples based on quality control measures, we have found no statistical relationships between *δ*^15^N and *δ*^34^S for sub-adults. Therefore, we have eliminated this discussion from the text all together, though we agree with the reviewer that there are more parsimonious explanations than that one we initially provided. We hope to explore the relationships in the future with additional datasets.

31. S6, P8, L2. Typo: pluralize ‘archaeologist’.

We have made this update in the text.

32. S7, P1, Last line. With respect to diet, I’m not sure you’ve convincingly demonstrated this with your sulfur isotope findings. Essentially, with respect to diet and *δ*^34^S, everything you’ve found is fairly (or very) speculative, which is fine, but I don’t know that this paper makes a clear case for how *δ*^34^S is going to be a major contributor to more nuanced paeleodiet studies in the area in the future. Points 2 (paragraph 3) and 3 (paragraph 4) in this section are primarily about provenance (and possibly secondarily about diet in point 2 – to the extent that people may have eaten different things in different places). Point 1 (paragraph 2) remains completely speculative in my view (see Comment 29). I don’t say this to downplay the importance of your work (it’s critical that we do these kinds of large scale studies in order to test the limits of what we can learn), but I would recommend tempering your language about the extent to which your data demonstrate the importance of sulfur isotope analyses for paleodietary work in the local area.

Based on this comment, and similar comments made by Reviewer 2, we have worked to reframe our concluding remarks with more tempered language recommended by Reviewer 1. In particular, we have emphasized that our interpretations of diet with respect to human sulfur data are preliminary and tentative. We also emphasize that, because systematically collected faunal baseline data are absent for most of the Eastern lowland sites discussed here, we are not able to confidently assess the inputs of freshwater (or potentially marine) resources. Our future research will address this issue. With respect to Comment 29 mentioned here, we have completely eliminated this from our conclusion (see Comment 29 above for more detail).

33. S7, P2, As per Comment 29, this is highly speculative and should not be presented as a finding for this research until relevant caveats (i.e, other explanations may be more likely) and contextualization (i.e., some kind of rational for why humans may have a unique *δ*^34^S TEF) are provided.

As per our response to Comment 29, we have eliminated this discussion from the paper entirely.

34. P4. Zooming out a bit here – if *δ*^34^S is telling us a lot about diet (not necessarily to say that it isn’t/can’t), how do you reconcile its dual role as a provenance proxy? In this context, what interpretive framework can be used to assess the importance of outlier *δ*^34^S as evidence for mobility, if we do consider diet to be a major factor. I think there needs to be more nuanced discussion of this kind of issue – which is lacking in a lot of literature – and this might be a nice place to do that.

We agree with the reviewer that this a discussion of the bio-social implications of stable isotope data is important to discuss. To address the reviewer’s comment, we have added a discussion of the potential implications of our results for understandings of diet and mobility among the Maya in the conclusion. At the macro-scale, *δ*^34^S data reflect differences in regional ecology and resource exploitation. Because stable isotope data are specific to an individual, they also lend themselves to exploring socially mediated dietary choices. When used in conjunction with other dietary proxies such as *δ*^13^C and *δ*^15^N, *δ*^34^S values could help to eliminate potential foods from individual dietary regimes, indicating dietary differences based on social or economic factors within and between communities. Regarding mobility, similar factors might influence individual movement or migration. When combined with other indicators of mobility, like strontium isotopes from tooth enamel that document origin at birth, sulfur isotopes can provide a powerful tool for examining migration across a person’s lifespan. Given that this article presents the first comprehensive application of sulfur isotope analysis in Maya archaeology, a detailed a more engagement with the theoretical literature is beyond the scope of the current study. We hope that future work can build upon our results and expand on the theoretical implication of sulfur isotopic analyses as part of a multi-proxy approach to examine diet and mobility in the future.

**Reviewer 2 Comments**

1. I also suggest the authors thoroughly proofread the writing to ensure correct scientific terminology is used, and make sure the correct studies are cited (I have spotted several mistakes below). For example, page 14, 2nd paragraph 9th line, it should be stable nitrogen isotope enrichment, not nitrogen enrichment.

We have made this update in the text, in addition to thoroughly proofreading our final submitted draft.

2. And page 16, last paragraph of section 5, should be “correlation between d15N and d34S values for subadults… “, instead of “d14N and d34S for sub-adults”. The word “values” need to be added throughout this section.

We have gone through the entire manuscript and added the word “values” as indicated by the reviewer.

3. Introduction: second line of second paragraph, “conclusive evidence” seems too strong, “compelling evidence”?

We have made this update in the text.

4. Intro, 2nd paragraph: the second “Tykot et al. 2002” reference in the second paragraph seems misplaced, or perhaps the authors meant they are building upon the works done by Tykot et al?

We have removed the section Tykot et al. 2002 citation from this paragraph to avoid confusion and have updated other citations that correspond more specifically to our points.

5. Last line of 3rd paragraph, other than Oelze et al. 2012 none of the study mentioned employed Sr isotope analysis, perhaps should rephrase the last sentence to “…other archaeological or isotope evidence”

We have made the suggested update in the text which has provided clarity, as indeed, the reviewer points out only the Oelze et al. 2012 paper used Sr analyses.

6. 4th paragraph 4th line: “…represents one of the largest…”

We have made this update in the text.

7. Last paragraph in intro: need to clarify the correlation between δ15N and δ34S values are observed among subadults.

Based on re-analysis of our dataset, after eliminating additional samples based on quality control measures, we have found no statistical relationships between N and S for sub-adults. Therefore, have eliminated this entire discussion from the paper and to not mention it in the introduction.

8. Intro: Need to clarify that some of the data are from previously published reports, and also mention published Sr data will be used.

We have clarified this in the introduction, specifically in the paragraph starting on Line 34.

9. Table 1 is excellent! It is really helpful for non-Mayan specialists like myself! Perhaps the authors can consider adding the number of samples analyzed from each period in this table?

This is a helpful suggestion and we have added the number of samples analyzed per period. Later in the text we note that one sample does not have an associated time period. Therefore, it does not appear in Table 1.

10. Section 2, 2nd paragraph, 4th line should be “the natural vegetations of the Maya lowlands…”

We have made this update in the text.

11. Page 5 first line, I would argue that ¹³C values of freshwater fish as “highly variable” instead of “typically more negative”.

We have made this update in the text.

12. Page 5 second paragraph, the reference of Cheung et al. 2019 is a mistake, that study did not look at S measurements.

We have made this update in the text by removing this erroneous citation.

13. Result section: perhaps showing the figures with significant patterns (like through time, rather than by region?)

This comment relates to Comments 19 and 20 below. To address this, we have included a figure of showing change in mean carbon values through time, in addition to table reporting mean *δ*^13^C, *δ*^15^N, and *δ*^34^S values through time. While there were statistically significant patterns in *δ*^34^S values through time, we do not include a figure of this in the main text since we do not find these patterns meaningful in terms of change in diet or migration. Rather, we think these patterns represent sampling biases, and describe this in the Supporting Information document (Se Text) as per the suggestion of Reviewer 1 (see their Comment 26 above).

14. Personally, I disagree that a low d34S values = “aquatic” sulfur isotope signature. d34S values in freshwater systems can vary greatly from region to region, some may have high d34S values.

We have updated our background section to include recently published faunal baseline data from Rand et al. (2021) that suggest a range of *δ*^34^S values for freshwater species, which are based on their location but also species-specific feeding habits (see Reviewer 1, Comment 13). However, we have also included a brief discussion based on a recent study by Guiry et al. (2021) that suggests that bone collagen in animals consuming wetland resources reflect high *δ*^15^N values and low *δ*^34^S signatures. We have in this case we have replaced the word “aquatic” with “low” to reflect these findings.

15. Page 5 3rd paragraph, the first sentence should be rewritten. I believe the authors are addressing the fractionation between consumer and source? In any case, 0.5 – 2.4‰ is a big range and not “slightly” different.

We have revised this sentence based on the language presented by Nehlich (2015). In particular, we point out that the -0.5 ‰ value the average with a ±2.4 ‰ standard deviation. While most researchers simply cite 0.5 ‰ as the trophic level enrichment factor, we feel that it is important to keep in mind that the standard deviation so as not to oversimplify sulfur fractionation. Much remains unknown about how enrichment works in regard to sulfur in the Maya region, so for the sake of transparency, we report standard deviation in the text.

Nehlich O. The application of sulphur isotope analyses in archaeological research: A review. Earth Sci. Rev. 2015; 142: 1-17. doi: 10.1016/j.earscirev.2014.12.002.

16. Section 3.1, 1000 sq km is not a “small” geographic area.

The text has been updated to reflect that the Eastern Maya lowlands is a circumscribed region of the Maya lowlands, and thus is it is subject to relatively constant and evenly distributed atmospheric conditions.

17. Figure 2 is a great map, but there isn’t much description in the text about how this model was produced. What parameters were involved? What statistical models and programs were used?

We have included more details in the text about how this map was created (Lines 286-289). The figure presents and isoscape model that was ArcMaP v. 8.0 using Inverse distance weighted (IDW) interpolation of expected *δ*^34^S values and average values for terrestrial fauna by site where available (Xunantunich, Pacbitun), with contours representing predicted values. The terrestrial faunal values were recently published by Rand and colleagues (2021) and were not available at the time of our initial submission. Therefore, we have updated Figure 2 to reflect these new values, though they are only slightly different form isocape model presented in our initial mansucript.

18. Section 3.2, perhaps the authors can briefly explain why the following sites are selected (what are the site selection criteria?).

We have included a few sentences describing the sampling strategy, which included consideration of geological/environmental zones, occupational histories of sites, and relatively good preservation contexts of burials.

19. Section 4.2 mentioned that %C and %N are used to evaluate bone collagen quality, but %C and %N are not showing in S1, please amend that.

These values appeared in our original S1 table, but we have double checked that they appear in our revised submission.

20. Table 3, is it possible to also add chronological info to this table?

We initially reported chronological trends in *δ*^13^C, *δ*^15^N, and *δ*^34^S in the supplemental documentation. We could not find a way to easily covey chronological and site-specific average values since not all time periods were represented at each site. To address the reviewer’s comment, however, we have included a table (Table 4) with chronological data that was originally in the supplemental section.

21. I recommend the authors show a figure comparing d13C values through time.

We originally included a figure comparing *δ*^13^C values through time in the Supporting Information document, but have added it to the manuscript as Figure 3.

22. As no faunal data is available, the discussion of trophic level seems inappropriate (section 5.1).

We have removed the discussion of trophic level and instead have updated our language to reflect the provenance of protein resources (in this case, terrestrial).

23. Section 5.1, I personally don’t think it is a good idea to correct infant nitrogen isotope enrichment (Reynard et al’s paper certainly did not recommend that) to match the values of the adults.

Based on this comment, and the fact that we did not correct isotope values for infants to match adults in this study, we removed the statement referenced by the reviewer.

24. Section 5.2, I do not follow the discussion about “among terrestrial faunal species….”, please expand on this, or even include the faunal data point on the graph.

Since we do not present faunal data in the paper, we have decided to remove this sentence to improve clarity in the text.

25. Table 4, to me, the mean of group1 and 2 are so close that it is almost within analytical error, and group 4 only consists of 2 datapoints, so in reality, there should really be 2 groups?

Based on the Editor’s comment (comment #1) we have re-analyzed the dataset, excluding additional samples with questionable quality control measures. The results have modified our interpretations of the sulfur groups. Instead of 4, we now suggest that there are two distinct that can be identified, which is in line with the reviewer’s observations. Group 1 consisted of Belize Valley and Belize Valley southern periphery sites (Table S19 and Figure S4). Group 2 included sites primary from the Vaca Plateau, with one site from the Belize Valley and one sites from the southern periphery. Group 1 has a significantly higher mean *δ*^34^S values compared to Group 2 (*t* = 6.66; *df* = 76; p > 0.001), general corresponding is g with geology. We estimate that mean *δ*^34^S values for individuals from Group 1 sites is 13.1 ± 0.5 at the 99% CI. Our estimate for Group 2 *δ*^34^S values is 10.7 ± 1.0 at the 99% CI. A possible third group may also exist, but because of the small sample size (*n*=2; Maya Mountain sites), statistical analyses could not be performed to clearly identify it as a distinct group. This information is supplied in the Supporting Information document in (S3 Text), where we also include tables and figures supporting these interpretations.

26. A figure showing correlation between the d13C and d34S values of the subadults could be interesting.

Based on re-analysis of our dataset, after eliminating additional samples based on quality control measures, we have found no statistical relationships between N and S for sub-adults. Therefore, we do not include this figure.

27. Without any faunal data, I think it is inappropriate to discuss dietary compositions in too much specificity.

We have revised our wording to reflect this suggestion. Instead of discussion specific trophic levels of food resources we categorize them generally by terrestrial animals vs. plants.

28. Page 16 last line (to page 17 first line), be careful with citations here, Vika 2009 and Fornander 2013 only look at S measurements and did not use s isotope values to supplement results from strontium isotope analysis.

We have updated the citations to correct this error.

29. Page 17, line 8, tropic should be trophic. However, as mentioned earlier, I don’t think with current data it is possible to discuss dietary compositions in such specificity.

We have made this update throughout the text. See Reviewer 2, Comment 27 above.

30. I think the comparison of d34S and 87Sr/86Sr values is a good idea! I wonder how the Sr measurements correlate with the geological map and isoscape model in figure 2?

Because we found that strontium and sulfur generally correlate for “local” individuals, the Sr data correspond relatively well to the isoscape model. That being said, strontium analyses have not been evenly applied across the study area (due in part to biases in the skeletal assemblage as well as in research intensity), and the addition of individuals from new sites are continually changing our interpretations of “local” signatures. While we do not discuss this specifically in the paper, we plan to conduct a larger study specifically focused on us the of sulfur and strontium data to look at movement across individual’s lives. Given that strontium in bone is often subject to diagenesis, we believe that sulfur might be a useful alternative to look at migration at the end of a person’s life.

31. While the d34S values of the infants are interesting, I am not fully convinced of these “correlations”. The samples come from a vast temporal and geographical contexts, given that no faunal data is available to monitor regional patterns, and that the subadult sample size is very small, I recommend the authors be more subtle about their interpretations.

As per Reviewer 2, Comment 26 above, we have eliminated this discussion based on a lack of correlations found after re-analysis.

32. Conclusion: I think the authors need to word the conclusion more carefully. As there is no faunal baseline data from the sites analysed, the authors need to tread very carefully and do not overinterpret their data. Freshwater fish especially, can have unpredictable/unexpected isotopic signature, therefore I don’t particular agree that d34S values can be used to say anything about the consumption of freshwater resources, at least not with current data. The second point is great, but kind of overlaps with the third point (did the outliers ate lots of non-local food or were they non-local themselves?). Perhaps the authors can rephrase this.

Based on this comment, as well as those from Reviewer 1 (Comments 32-34), we have reframed our concluding remarks. Specifically, we have emphasized that our interpretations of human sulfur data are preliminary and tentative. We also emphasize that, because systematically collected faunal baseline data are absent for most of the Eastern lowland sites discussed here, we are not able to confidently assess the inputs of freshwater resources. Our future research will address this issue. Based on this, comment, we also combined our second and third points into a single paragraph about the great potential this study demonstrates for using sulfur isotopes in mobility and migration studies in the Maya lowlands.
