# Supplementary material for: Sulfur isotopes as a proxy for human diet and mobility from the preclassic through colonial periods in the Eastern Maya lowlands
Source: PLoS One. 2021 Aug 12;16(8):e0254992. doi: 10.1371/journal.pone.0254992 (PMC8360522; doi:10.1371/journal.pone.0254992)
Supplement: Attachment — Submitted filename: Response to Reviewers - Ebert et al_R2.docx [file pone.0254992.s008.docx]

**Response to Reviewers – Revision 2**

**Editor Comments**

1. *As far as preservation criteria are concerned, I have spotted one sample (MARC 2571 line 81 of excel table 1 S1) with a C:N ratio of 3.7 that should be excluded from further interpretation for the sake of consistency with the recommendation of De Niro (1985).*

We have eliminated this additional sample from our statistical analyses following conventions set by DeNiro (1985). This is reflected in Table S1. Our updated analyses are presented in the results section of the manuscript and in the supplemental information (S3 Text). Our revised statistical analyses did not change the interpretations of the dataset and temporal/regional trends presented in our first revisions.

2. *About literature citation, I am not sure that the reference to O’Connell et al. (2012) is making sense to keep if you finally favour a more acknowledged TEF range of values unless you add “but see O’Connell et al., 2012” to mention possible debated on that point. Along the same lines, to my knowledge, Hedges and Reynard (2007) did not demonstrate a higher number of trophic levels in marine ecosystems. Pending the revision of the above points, the manuscript could be considered for publication.*

We have removed the O’Connell et al. (2012) citation from the paper as we have formed our discussion of data around a TEF of ~+3 to +5 ‰. We have also removed the Hedges and Reynard (2007) citation from the paper. After considering the editor’s comments, we felt that we had incorrectly used it in several placed. In the instance referenced by the editor, we have cited a study by Harriston and Harriston (1993) that specifically discusses trophic structures in terrestrial vs. aquatic ecosystems (citation 53, line 150). We also replaced the Hedges and Reynard (2007) citation at its first appearance in the manuscript (citation 31, line 122) with a more appropriate citation that discusses the use of isotope analyses of bone collagen to track diet during the last several years of an individual’s life.
