# Supplementary material for: Sulfur isotopes as a proxy for human diet and mobility from the preclassic through colonial periods in the Eastern Maya lowlands
Source: PLoS One. 2021 Aug 12;16(8):e0254992. doi: 10.1371/journal.pone.0254992 (PMC8360522; doi:10.1371/journal.pone.0254992)
Supplement: Attachment — Submitted filename: Reviewer Comments - Guiry March 22 2021.docx [file pone.0254992.s006.docx]

**Title Reviewed:** *Applying Sulfur Isotopes to Paleodietary Reconstruction and Human Mobility from the Preclassic through Colonial Periods in the Eastern Maya Lowlands*

**Recommendation:** Minor-moderate revisions

Dear Dr. Drucker,

Thank you kindly for the invitation to review “Applying Sulfur Isotopes to Paleodietary Reconstruction and Human Mobility from the Preclassic through Colonial Periods in the Eastern Maya Lowlands*”* for *PLoS One* (manuscript number: PONE-D-21-05019). This manuscript presents results from stable carbon, nitrogen, and sulfur isotope analyses of collagen extracted for human bone and compares these isotopic compositions between samples grouped by time, location, and age to assess Mayan mobility and diet. While in many cases interpretations are speculative, results appear to confirm prior expectations based on regional geology and what has been learned from previous paleodietary studies.

The paper is well very written and generally well researched/argued and I believe it will be of interest to archaeologists working in the region. For this reason, I would support the paper’s acceptance for publication following consideration of the comments listed below. Please note that comments 12 and 29 are of greater importance. These comments are primarily geared towards improving interpretive context and clarity and, in a few cases, tempering/adjusting language around interpretations in light of potential uncertainties that appear to have been overlooked by the authors.

If you have any questions about these comments or my recommendation, please do not hesitate to contact me directly.

Sincerely,

Dr. Eric Guiry

School of Archaeology and Ancient History

University of Leicester

**Reviewer Comments**

This manuscript was a pleasure to read. It is well written and I would like to commend the authors on what appears to be a generally well designed and executed study. That said, I recommend the following changes, which are aimed at enhancing the paper’s isotopic background and clarifying language around some interpretations.

1. In future submissions please include line numbers so that peer reviewers can spend less time pinpointing the lines to which they’re referring. In the following comments: S = section number; P = paragraph number within that section; L = line number(s) within that paragraph.
2. S2, P2, L7. You may wish to change this slightly as it is technically more correct to say “less negative” rather than “more positive” when referring to negative values.
3. S2, P2, L9. This is perhaps a bit simplistic – C_3_ environments have a tremendous range of *δ*^13^C variation, which can lead to systematic variation in human and animal *δ*^13^C. For instance, major axes of *δ*^13^C variation include structural differences among C_3_ environments, such canopy density (Drucker, et al. 2008; van der Merwe and Medina 1991), or dietary foci on differ plant tissues (i.e., vegetation vs. mast/nuts/seeds), which can lead to multi ‰ offsets in consumers (Guiry, et al. 2020). Given the importance of *δ*^13^C in regional diet interpretations, and the potential significance of forest ecosystems in the area, these kinds of potential sources of *δ*^13^C variation would seem relevant to mention.
4. S2, P2, Last line. Again, a bit more nuance would be useful here. In archaeology we tend to vastly oversimplify variation in marine resource *δ*^13^C, and this can lead to missed opportunities for understanding past diets. The bone collagen *δ*^13^C of aquatic species can span 10-15‰, based on the type of marine environments, and for many economically important fish *δ*^13^C can be very low indeed (i.e., easily overlapping with C_3_ diets). The ecological literature (although often focusing on other tissues) is a good place to start to get a better sense of this (e.g., Satterfield and Finney 2002). Not that you’d need to expand much here, but some acknowledgment of these potential issues would add interpretive transparency for readers with less background in biogeochemistry.
5. S2, P2, Last line. Wasn’t Chisholm, et al. (1982) first to look at this in an archaeological context?
6. S2, P3, L2. The 6‰ trophic enrichment factor (TEF) estimate is considered by many to be extreme. It’s a hypothetical estimate based on stacked uncertainties from combining inter-tissue offsets. Are you weighing this TEF on equal footing with the much more widely acknowledged and studied 3-4‰ estimates?
7. S2, P3, L4-6. Again some more nuance here might be helpful. Like *δ*^13^C, *δ*^15^N baselines in marine environments are highly context dependent (but at least for *δ*^15^N, there’s a really good review out there: (Sigman, et al. 2009)). You’re explanation for why marine foods lead to high *δ*^15^N in consumers is not quite correct. The reason that *δ*^15^N is often assumed to be higher in marine foods than terrestrials foods (by archaeologists anyhow) has more to do with trophic level than it does baselines. Marine ecosystems can have up to 5 trophic levels while terrestrial systems top out at 3. This leads to much higher *δ*^15^N for people eating of marine animal protein from TLs 3-5. Consumption of marine foods at TLs 1 or 2 won’t necessarily lead to higher bone collagen *δ*^15^N.
8. S2, P3, L7-8. Archaeologists widely assume that freshwater fish *δ*^13^C should be lower, and this is certainly true for select species in some areas (we even get bone collagen *δ*^13^C in the –30s in some regions), but it not necessary the case everywhere. Guiry 2019, which you cite here, is actually cautioning archaeologists to pay more attention to this variation, especially potential sources of higher *δ*^13^C in freshwater foods – which can happen when fish are sourced from certain places like nearshore areas, higher productivity ecosystems, and potentially areas where aquatic carbon budgets may be influenced by CO_2_ subsidies from ancient marine sedimentary substrates. If you don’t have data from archaeological fish in hand, you may want to explore whether it’s likely that relevant fish taxa in the area would likely have had higher or lower *δ*^13^C based on local geography/ecology of applicable watersheds. Also, rather than being tucked into a line about *δ*^15^N, potential issues about variation in freshwater resources *δ*^13^C should be dealt with in the preceding paragraph, and given some dedicated attention (again, especially given the importance of *δ*^13^C for interpreting overall trends in diet in the region).
9. S2, P3, Last line. The potential importance of agricultural impacts on *δ*^15^N should also be acknowledged, especially in this region. For an excellent review on this, see Szpak (2014). It may also be worth considering the potential impacts of agriculture on *δ*^34^S as well (Szpak, et al. 2019).
10. S2. P4, L10-13. This has now been empirically demonstrated within a modern experimental context using bone collagen (Guiry and Szpak 2020).
11. S2. P4, L13-15. I’m not sure to what extent estuarine resources could be relevant here, but it might also be worth noting that some ‘freshwater’ resources from areas that receive minimal seawater inputs can still appear ‘marine’ in their *δ*^34^S. This is because allochthonous seawater S inputs, due to the much higher S content of seawater relative to freshwater, overwhelms autochthonous freshwater S sources (Fry and Chumchal 2011). This can mean that species you’d assume were freshwater, will contribute a ‘marine’ *δ*^34^S signal to diet.
12. Something you begin to allude to here, but which can (and I’d argue really should) be considered in more detail is the potential importance of sulfide-derived S in wetland resources. A recent paper has demonstrated a very large impact of consuming sulfide tolerant plants (or plants in areas where there is more sulfate from reoxidised sulfide) on consumer bone collagen *δ*^34^S (Guiry, et al. 2021). While this archaeological study was focusing on costal marshes, the principles at play may also apply at inland wetlands as well (early data from (Cornwell, et al. 1995) suggests this). As outlined in the 2021 paper, the underlying mechanisms that leads to lower *δ*^34^S in wetlands (anoxia) may also result in higher *δ*^15^N (due to coupled nitrification-denitrification in marsh adapted rhizomes) for affected plants and their consumers. This means that you could be able to assess the potential for this kind of resource consumption in your samples with the data you already have. My feeling though is that the broad scale diet of humans might serve to obscure patterns, but this may, at least, help you assess what is going on with your low *δ*^34^S outlier (see Comment 21).
13. S3.1, P1, Last line. This seem like a slight over generalization. As per Comment 12, the importance of wetland resource could have a meaningful impact and you could do more to unpack this or at least acknowledge that fact that large uncertainties remain in your ability to anticipate/untangle the relative importance of geological vs. dietary (between the poles of marine- and sulphide-influenced *δ*^34^S) source of sulfur isotope variation. As it stands, based on this statement, I think readers without a strong background could misunderstand the degree of confidence we can have in these kinds of data.
14. S3.2. You go into extensive site-by-site detail here, which is good. Reading the paper, I felt that this was a bit out of balance with the detail/attention you give to isotope ecology. I point this out here to support the above and below suggestions for giving closer consideration to sources of uncertainty in isotopic interpretations in general. These may be critical in terms of interpretive transparency for many of your readers who are not isotope specialists, to help them really grasp the limits of what these data can mean.
15. S4.2, P1, L3. I’m not sure you’re citing Szpak correctly here. The paper that appears in the ‘literature cited’ section is about reporting and calibration but you appear to be referring to procedures for collagen extraction here. Do you to cite Szpak’s NaOH study? (Szpak, et al. 2017)
16. S4.2, P3, L2. I’d encourage you to report C:N to the second decimal place here and elsewhere. Note that while precision/accuracy of the %C and % N measurements that are used to calculate C:N ratios are typically not accurate to the second, or even first, decimal place, the ratio between %C and %N definitely should be. Moreover, the second decimal place matters here – meaningful isotopic differences can occur due to contamination that is detectable even at this minute scale of variation in C:N.
17. S4.2, P3. I do not have a problem with this, but technically the QC criteria you’re reporting here are part of the results and journals often require this kind of information to be placed in the results section rather than methods. One could say the same for the standard uncertainty calculation in the preceding paragraph (but for some reason, and in contrast to my comment here on collagen QC, these are typically reported in the analytical methods section).
18. Table 3. Are these “*n*=” values for the *δ*^34^S data? If so, why does Pacbitun not have a SD reported? If, not, please report these here. Also, in Section 5, Paragraph 1, you note that relevant citations for isotopic data are given in the SI. Why cite then Ebery 2019 under Table 3 as well then?
19. S5.1, P2, Last few lines. Trophic level is only one of several archaeologically meaningful variables that could be at play here. There’s a lot more you could consider in this context in terms of unpacking these other sources of *δ*^15^N – not least the potential influence of agriculture on N cycling/soil-plant *δ*^15^N as per Comment 9. Readers may wonder why these issues/alternatives are not discussed.
20. S5.1, P3, L3. Be careful in your terminology here with phrases like “nitrogen values”. While I do understand what you mean, this term could refer to either elemental of isotopic compositions and can therefore lead to reader confusion/uncertainty.
21. S5.1, P4, L3. Be sure to define your acronyms (IQR), even if they should be obvious.
22. S5.2, P1, L14. I’ve seen this in other papers and have always wondered what empirical basis it has (i.e., the idea that a sample falling a certain number of SDs away from a group is statistically meaningful). I may just be less well-versed in this approach to finding meaning in a dataset. But if I am left wondering about this question, other readers may be too and it might be worth very briefly unpacking the math or modelling that this approach is based on.
23. S5.2, P1, L4-to-end-of-paragraph. It would worth re-exploring these lower *δ*^34^S in the context of Comment 12. If the lower *δ*^34^S co-occur with higher *δ*^15^N, it could be stronger evidence that sulfide-derived S is behind these low values (indicating anoxic/wetland production). Also, I would be carful in offering bacterial sulfate reduction alone as an explanation. The product of this is sulfide, which is toxic to most plants. Low *δ*^34^S from sulfide would not automatically be reintroduced to a food web – one of two things still needs to happen: either, 1) reoxidation of sulfide-S to sulfate, which is then somehow injected back in a food web; or, 2) presence of sulfide tolerant plants (i.e., with symbiotic relationships with sulfur-oxidizing bacteria or that may themselves be chemolithotrophic). The latter is a question mark for sure, but these may be more prevalent in wet lands. In any case, the fact that bacterial sulfate reduction creates a source of isotopically light S, does not in and of itself provide a plausible source for low *δ*^34^S values.
24. S5.2, P3, L2. Here and elsewhere, please add “*n*=” statements in brackets for each group you compare. Please also add the mean and SD for isotope compositions in each group in brackets. This saves a lot of work for the reader.
25. S5.2, P3, Last line. No worries either way here, but if geographical bias was a likely finding from the get-go, one could ask why make these temporal comparisons? Might they be better positioned in the SI?
26. S6, P4, Last two lines. Following on Comment 9, there are potentially a lot of other reasons a play here, like N cycle openness and fertilization, which can lead to multi ‰ increases in *δ*^15^N of consumer collagen through time (Guiry, et al. 2018). It would at least be worth considering the potential impacts of agriculture, in addition to land clearance.
27. S6, P5, L3-6. Our baselines for marine fish are fairly biased to select European species, particularly from an archaeological perspective. There are marine ecosystems (including in the Gulf of Mexico) where food webs are heavily influence by sulphide-derived S (e.g., Chasar, et al. 2005) – this is especially the case in some seagrass areas (Fry, et al. 1982; Holmer and Hasler-Sheetal 2014), due to the importance (like in wetlands) of sulfide-derived S in primary production. I’m not sure of the extent to which seagrasses are ecologically important in your broader study area, but it is worth bearing in mind that fully marine fish can have very non-‘marine’ *δ*^34^S.
28. S6, P5, Last line. Can you outline why consumption of domesticates would lead to a higher *δ*^15^N? There are plenty of reason (e.g., Comments 9 and 26), but given that these animals are presumably of a similar trophic level to locally available wild animals, readers may be confused.
29. S6, P7, L 14-20ish. The suggestion of this being evidence for a TEF for *δ*^34^S is really hypothetical and speculative. This is not an issue in and of itself, but there could be any number of reasons for the differences your seeing here, some of which (like there being a real dietary difference between infants and adults) seem more plausible to me than the idea that humans have some kind of unique fractionation for S isotopes. Bone collagen S comes from methionine, which is an essential amino acid for humans and, without offering some kind of biochemical explanation or pathway for what could plausibly explain such a TEF, it really seems like this is beyond the pale, or just too speculative to foreground like this here (let alone calling it a ‘tentative conclusion’). If you’re going to include this discussion, please add a strong caveat noting that: 1) there are/may be potentially more plausible/parsimonious explanations (e.g., diet differed); and 2) in the context of existing literature, some plausible explanation for how/why humans could have a substantial sulfur isotope TEF while other mammal species do not.
30. S6, P8, L2. Typo: pluralize ‘archaeologist’.
31. S7, P1, Last line. With respect to diet, I’m not sure you’ve convincingly demonstrated this with your sulfur isotope findings. Essentially, with respect to diet and *δ*^34^S, everything you’ve found is fairly (or very) speculative, which is fine, but I don’t know that this paper makes a clear case for how *δ*^34^S is going to be a major contributor to more nuanced paeleodiet studies in the area in the future. Points 2 (paragraph 3) and 3 (paragraph 4) in this section are primarily about provenance (and possibly secondarily about diet in point 2 – to the extent that people may have eaten different things in different places). Point 1 (paragraph 2) remains completely speculative in my view (see Comment 29). I don’t say this to downplay the importance of your work (it’s critical that we do these kinds of large scale studies in order to test the limits of what we can learn), but I would recommend tempering your language about the extent to which your data demonstrate the importance of sulfur isotope analyses for paleodietary work in the local area.
32. S7, P2, As per Comment 29, this is highly speculative and should not be presented as a finding for this research until relevant caveats (i.e, other explanations may be more likely) and contextualization (i.e., some kind of rational for why humans may have a unique *δ*^34^S TEF) are provided.
33. P4. Zooming out a bit here – if *δ*^34^S is telling us a lot about diet (not necessarily to say that it isn’t/can’t), how do you reconcile its dual role as a provenance proxy? In this context, what interpretive framework can be used to assess the importance of outlier *δ*^34^S as evidence for mobility, if we do consider diet to be a major factor. I think there needs to be more nuanced discussion of this kind of issue – which is lacking in a lot of literature – and this might be a nice place to do that.

**References**

Chasar LC, Chanton JP, Koenig CC, Coleman FC. 2005. Evaluating the effect of environmental disturbance on the trophic structure of Florida Bay, U.S.A.: Multiple stable isotope analyses of contemporary and historical specimens. Limnology and Oceanography 50(4):1059-1072.

Chisholm BS, Nelson DE, Schwarcz HP. 1982. Stable-carbon isotope ratios as a measure of marine versus terrestrial protein in ancient diets. Science 216(4550):1131-1132.

Cornwell JC, Stevenson JC, Neill C. 1995. Biogeochemieal origin of δ34S isotopic signatures in a prairie marsh. Canadian Journal of Fisheries and Aquatic Sciences 52(8):1816-1820.

Drucker DG, Bridault A, Hobson KA, Szuma E, Bocherens H. 2008. Can carbon-13 in large herbivores reflect the canopy effect in temperate and boreal ecosystems? Evidence from modern and ancient ungulates. Palaeogeography, Palaeoclimatology, Palaeoecology 266(1–2):69-82.

Fry B, Chumchal MM. 2011. Sulfur stable isotope indicators of residency in estuarine fish. Limnology and Oceanography 56(5):1563-1576.

Fry B, Scalan RS, Winters JK, Parker PL. 1982. Sulphur uptake by salt grasses, mangroves, and seagrasses in anaerobic sediments. Geochimica et Cosmochimica Acta 46(6):1121-1124.

Guiry E, Noël S, Fowler J. 2021. Archaeological herbivore δ13C and δ34S provide a marker for saltmarsh use and new insights into the process of 15N-enrichment in coastal plants. Journal of Archaeological Science 125:105295.

Guiry E, Orchard TJ, Needs-Howarth S, Szpak P. 2020. Isotopic Evidence for Garden Hunting and Resource Depression in the Late Woodland of Northeastern North America. American Antiquity 86(1):90-110.

Guiry E, Szpak P. 2020. Seaweed-eating sheep show that δ^34^S evidence for marine diets can be fully masked by sea spray effects. Rapid Communications in Mass Spectrometry 34:e8868. .

Guiry EJ, Beglane F, Szpak P, Schulting R, McCormick F, Richards MP. 2018. Anthropogenic changes to the Holocene nitrogen cycle in Ireland. Science Advances 4(6):eaas9383.

Holmer M, Hasler-Sheetal H. 2014. Sulfide intrusion in seagrasses assessed by stable sulfur isotopes—a synthesis of current results. Frontiers in Marine Science 1(64).

Satterfield FR, Finney BP. 2002. Stable isotope analysis of Pacific salmon: insight into trophic status and oceanographic conditions over the last 30 years. Progress in oceanography 53(2):231-246.

Sigman D, Karsh K, Casciotti K. 2009. Ocean process tracers: Nitrogen isotopes in the ocean. In: Steele JH, Turekian KK, Thorpe SA, editors. Encyclopedia of Ocean Science. 2 ed. San Diego: Academic Press. p 4139-4152.

Szpak P. 2014. Complexities of nitrogen isotope biogeochemistry in plant-soil systems: implications for the study of ancient agricultural and animal management practices. Frontiers in Plant Science 5.

Szpak P, Krippner K, Richards M. 2017. Effects of Sodium Hydroxide Treatment and Ultrafiltration on the Removal of Humic Contaminants from Archaeological Bone. International Journal of Osteoarchaeology 27(6):1070-1077.

Szpak P, Longstaffe FJ, Macdonald R, Millaire J-F, White CD, Richards MP. 2019. Plant sulfur isotopic compositions are altered by marine fertilizers. Archaeological and Anthropological Sciences 11(6):2989-2999.

van der Merwe NJ, Medina E. 1991. The canopy effect, carbon isotope ratios and foodwebs in Amazonia. Journal of Archaeological Science 18(3):249-259.
